# Early-warning surveillance of West Nile and Usutu viruses in wetland and larval habitat waters and in mosquito excreta using digital PCR

**DOI:** 10.1371/journal.pntd.0014799

**Published:** 2026-10-06

**Authors:** Julien Mocq, Julie Raymond, Karine Bolloré, Anaïs Fossot, Rachel Beaubaton, Alexandre Lepeule, Cécile Gruet, Franz Durandet, Jeanne Hanin, Guillaume Lacour, Albin Fontaine, Olivier Courot, Antoine Mignotte, Yannick Simonin

**Affiliations:** 1 ALTOPICTUS, Pérols, France; 2 IAGE, Ingénierie et Analyse en Génétique Environnementale, Montpellier, France; 3 GIS OBEPINE+, Sorbonne Université, Paris, France; 4 UMR, INSERM, Université de Montpellier, Pathogenesis and Control of Chronic and Emerging Infections, Montpellier, France; 5 Unité de Parasitologie et Entomologie, Département des Maladies Infectieuses, Institut de Recherche Biomédicale des Armées, Marseille, France; 6 Aix Marseille Université, IRD, AP-HM, SSA, UMR Vecteurs – Infections Tropicales et Méditerranéennes (VITROME), IHU – Méditerranée Infection, Marseille, France; Beijing Children's Hospital Capital Medical University, CHINA

## Abstract

West Nile virus (WNV) and Usutu virus (USUV) are mainly avian mosquito-borne pathogens, increasingly cause human and equine disease in temperate regions. Mostly asymptomatic, current surveillance in France is reactive, based on clinical and veterinary reports, delaying signals for vector control and preventative interventions. We implemented in Southern France, in 2024 and 2025, a proactive surveillance methodology combining molecular xenomonitoring of mosquito excreta and environmental water detection of viral RNA in wetlands and mosquito larval habitat, through a multiplex reverse transcription digital PCR workflow. The analytics process was characterised through the limits of blank, detection, and quantification. Low-level positives were confirmed by Sanger sequencing. WNV and USUV RNA repeated detections revealed previously unrecognized, cryptic circulations up to 19 weeks before the first WNV human cases and equine alert. Environmental surveillance represents a paradigm shift for early detection of emerging *Culex*-borne arboviruses and a key enabler of proactive and effective One Health responses.

## Author summary

West Nile virus (WNV) and Usutu virus (USUV) are mosquito-borne flaviviruses maintained in enzootic cycles involving birds and mosquitoes, with incidental infections in humans and horses. Because most infections are asymptomatic, viral circulation often remains undetected until the onset of clinical cases, limiting the timeliness of current surveillance systems.

In this study, we developed an integrated environmental surveillance methodology in southern France, based on the molecular detection of viral RNA in mosquito excreta and environmental water samples collected from wetlands and larval habitats. Using a highly sensitive molecular method, we repeatedly detected both viruses during the 2024 and 2025 transmission seasons.

Importantly, viral RNA was identified several weeks prior to the first reported human and equine cases, highlighting the capacity of these matrices to capture early transmission. signals.

These findings demonstrate that environmental surveillance can uncover cryptic viral circulation not detected by conventional reactive surveillance systems.

Overall, combining water-based monitoring with mosquito faeces xenomonitoring provides a complementary and proactive surveillance strategy, with strong potential to inform earlier public health interventions and targeted vector control.

## Introduction

West Nile virus (WNV) and Usutu virus (USUV) are emerging threats in temperate regions, as climate warming, urbanization and biodiversity loss collectively alter the ecology, abundance and behaviour of mosquito vectors and avian reservoir hosts, promoting viral geographic expansion and increasing outbreak frequency [1–3]. These avian [4] neurotropic orthoflaviviruses are primarily transmitted by ornithophilic *Culex* mosquitoes [5,6], predominantly *Culex pipiens* in Europe [7]. Non-avian vertebrates, such as humans and horses, may be infected through infected mosquitoes taking blood meals, but are epidemiologically incidental “dead-end” hosts with typically low viremia, unable to infect subsequent biting mosquitoes [8]. Approximately 80% of WNV-infected horses or humans are asymptomatic, but clinical manifestations range from influenza-like symptoms to severe neuroinvasive diseases [9]. USUV, usually causing mild or no symptoms [10], can trigger serious neurological complications, particularly in immunocompromised individuals [11]. To date, no human antiviral treatments or vaccines exist for both viruses [12].

Since the 1990s, recurrent WNV-epidemics occurred in southern and eastern Europe [7] while USUV spread across Europe in the early 2000s [11] since its emergence in 1996 [13]. In France, the first human and equine WNV cases were identified in 1962 in the Camargue region [14], and the first human USUV case in 2016 in Montpellier [15]. The past few years, alongside multi-compartment USUV detections [16–18^]^, France experienced major WNV outbreaks of autochthonous human and equine cases, in both historically and newly affected regions [19], highlighting active viral circulation and ongoing northern and westward expansion [20–22^]^.

The surveillance and control of WNV and USUV viruses in France remain limited by structural, financial, and operational constraints, impeding early detection and rapid intervention. Unlike for *Aedes*-borne viruses, France lacks a comprehensive risk-prevention and management framework for *Culex*-transmitted WNV and USUV. As a notifiable disease at the EU level, WNV surveillance typically begins after the declaration of a confirmed equine or human case. Similarly, incidental USUV antibody detections during WNV serological screening suggest broader circulation than currently recognized [5,15]. Consequently, conventional surveillance approaches may provide late indicators of viral presence [23]. Against this backdrop of potentially undetected, low-level viral circulation, current responses to confirmed or suspected WNV risk often rely on emergency adulticide applications or untargeted preventive larvicide treatments, at a cost reaching millions [24]. Improving early, sensitive detection of WNV and USUV could therefore support more timely and targeted vector control interventions, while reducing reliance on costly, reactive measures.

Molecular Xenomonitoring (MX) offers a non-invasive, cost-effective, and integrative alternative to the labour-intensive mosquito pooling and dissection, addressing the urgent need for timely arbovirus surveillance. It relies on detection of viral RNA in mosquito excreta [25], only detectable in effectively infectious mosquitoes with disseminated infection [25]. Viral detection in mosquito excreta enables real-time surveillance of WNV and USUV circulation, but that viral detection in mosquito excreta provides evidence of ongoing viral circulation rather than a predictive signal of virus emergence [23]. However, the analysis of environmental matrices, particularly water, is a promising strategy of early detection of WNV and USUV circulation in avian and mosquito compartments [16,26]. Virus particles in water can originate from oviposition fluids excreted by infected mosquitoes –potentially infecting larvae by vertical transmission [27]–, dead infected mosquitoes, infected larvae, or bird faeces. Indeed, WNV and USUV genomes are present in the digestive systems of infected birds few days post-infection [28] and excreted into the environment in the faeces [16,28]. While the presence of viral genomes does not necessarily imply the presence of an infectious virus, its detection in environmental matrices may serve as an early warning signal to trigger reinforced surveillance and vector control operations, as demonstrated by the detection of viral RNA in wastewater prior to the onset of symptomatic cases [29]. Anticipating the risk emergence currently requires the quantification of the temporal lag between environmental detections of WNV or USUV and the onset of human or animal cases.

To improve early detection of WNV and USUV, we evaluated an innovative approach combining extensive sampling with molecular analysis of mosquito excreta and waters from potential larval development sites and wetlands, as adults and larvae habitats. Viral genomes are expected to be detected first in water during spring, following their introduction through excretion of infected migratory birds for WNV –as its endemicity in France is currently not firmly established– or infected migratory or resident birds for USUV, before the onset of mosquito activity and the biting season. Then, as mosquito activity increases and enzootic transmission cycles become established, we expected to detect the viruses in local mosquito populations involved in these ornithophilic cycles, preceding spillover events and, consequently, the occurrence of equine or human cases–hereinafter defined as detection of symptomatic or asymptomatic WNV/USUV cases in human or horses– and subsequent corresponding alerts –in the Ministry of Health’s information system dedicated to the prevention of vector-borne diseases (SI-LAV) platform [30] for human cases, and in the epidemiological surveillance network for equine diseases (RESPE) platform [31], in connection with the French National Reference Laboratory (LNR) for equine alert. We developed and validated a highly sensitive multiplex digital PCR assay for WNV, USUV, and mosquito-derived nucleic acids, with optimized extraction protocols for both matrices. In 2024, field evaluations in areas of known or suspected viral circulation aimed to optimize detection systems and establish proof of concept. In 2025, the method was deployed regionally to assess its performance and relevance for arboviral surveillance.

## Methods

### Investigated matrices

#### Wetland and artificial larval habitat water collection.

Environmental water sampling was carried out to detect viral RNA traces in wetland and larval habitats potentially harbouring infected avian and mosquito populations (Fig 1B). To maximise the chances of detection in wetlands, ecologically and epidemiologically relevant environments for potential WNV/USUV introduction and circulation were prioritized (S1 Table): avian biodiversity hotspots and congregation sites such as ornithological natural reserve, zoological parks [16], nesting and migratory stopover wetlands for wild birds and favourable *Culex* habitats [32], sites with a documented history of WNV/USUV circulation, sites with confirmed human or animal cases, or prior detections in mosquitoes in Occitania [16,33,34]. In 2024, surveillance efforts were intensified around equestrian facilities during WNV outbreak in equine population in Occitania [35]. These facilities are often located within or adjacent to wetland ecosystems (e.g., Camargue Regional Natural Park), bringing together domestic horses, resident and migratory bird populations, and dense *Culex* populations, creating key ecological interfaces conducive to arbovirus transmission. Wetland water was collected in sterilized 1-liter polypropylene bottles throughout the entire water column, with minimum sediment resuspension.

**Fig 1:**
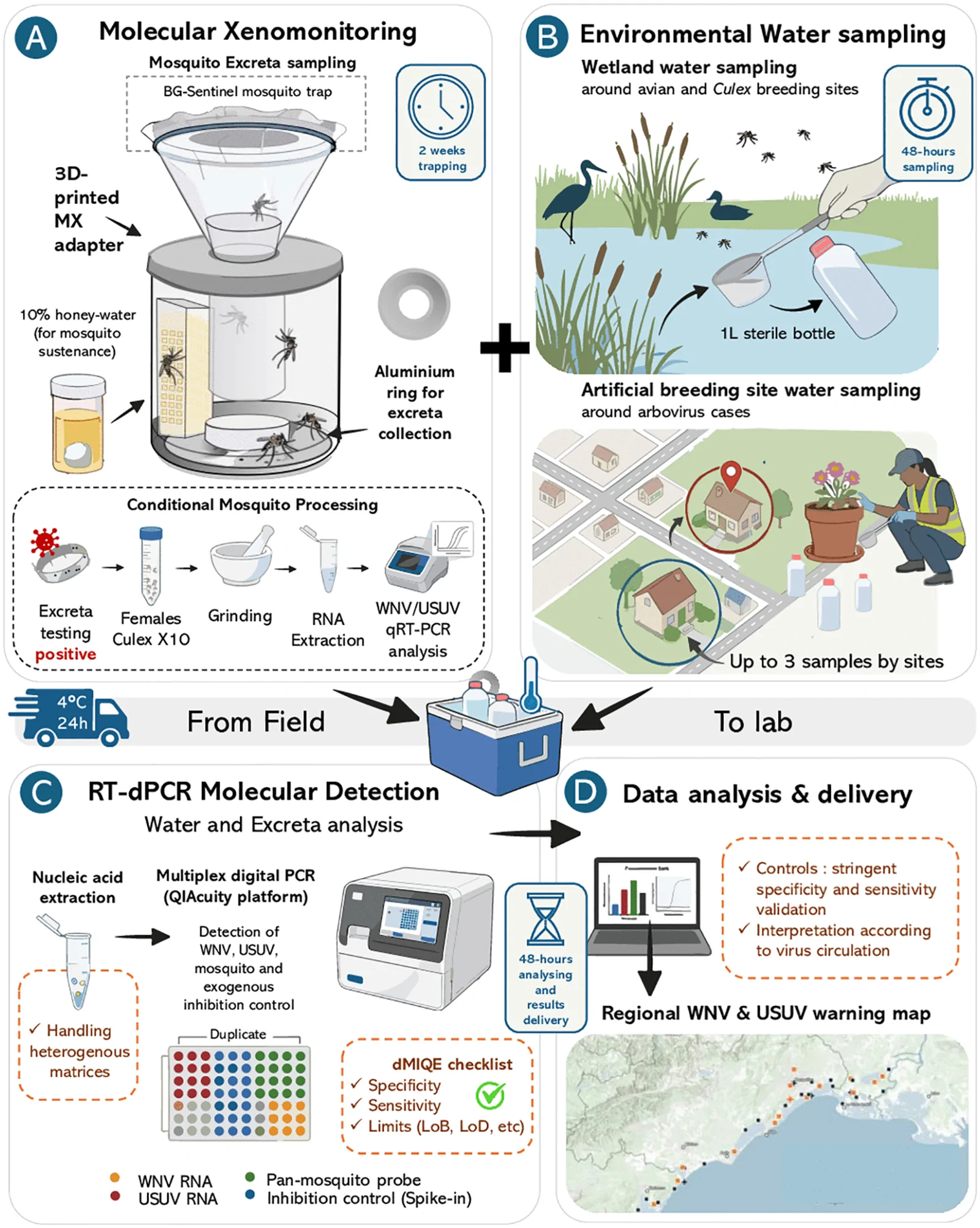
Early warning environmental surveillance workflow for WNV and USUV in 2024 and 2025 in Occitania, France. The figure was created primarily by us using Adobe Illustrator, with limited assistance from the FigureLabs AI tool (https://figurelabs.ai/terms).

Waters from artificial larval habitats (e.g., rainwater storage tanks) were mainly collected in urban areas during vector control operations mandated by the Regional Health Agency in Occitania, around confirmed local arbovirus cases. Targeted entomological investigations involved door-to-door inspections to eliminate potential and active *Culex* and *Aedes* mosquito larval habitats. These interventions provided opportunities to collect water samplings during attested arboviral circulation, in sterilized 1-liter polypropylene bottles. Samples were kept at 4 °C during transport, delivered at the laboratory within 48 hours and stored at 4°C until nucleic acid extraction.

#### Mosquito excreta.

The MX method was used to detect viral RNA traces in mosquito excreta, adapted and optimized for field surveillance (Fig 1A). Mosquitoes were trapped using BG-Pro or BG-Sentinel traps (Biogents, Germany) fitted with 3D-printed MX adapters [23]. Inside each adapter, a 30ml-capacity feeder with a 10% honey water-soaked cotton ball provided sustenance for trapped mosquitoes. A10 µm-thick aluminium foil ring (shiny side up) placed at the bottom of the adapter collected excreta and facilitated its recovery. After collection, the aluminium rings containing mosquito excreta (thereafter MX samples) were stored at −80 °C until further processing for RT-dPCR analysis.

Traps with MX were ideally installed few meters from a wetland sampling point, such as every short-term sampling battery-powered traps, allowing placement even in remote areas. However, for longer-term sampling traps requiring mains outlet, the distance between the trap and the closest wetland sampling site ranged from 5m to 1.9km. These traps were installed close to buildings, which were themselves surrounded by wetlands, or agricultural lands with close waterbodies (S1 Table).

Following a positive detection in mosquito excreta by RT-dPCR, all Culex females collected from the corresponding trap were retrieved and pooled by groups of 10 individuals. Viral RNA was extracted from these pools [36] and tested independently for WNV and USUV using a duplex qRT-PCR assay [37], as part of the complementary entomological investigation

### Field protocols

#### 2024 operational proof of concept.

Sampling was conducted from 15 April to 23 October 2024, corresponding to the active season of *Culex* spp. in southern France [38]. Based on hereinabove ecologically and epidemiologically relevant site types, 48 sampling sites were selected across 13 municipalities within the Gard and Hérault departments (Fig 2A; S2A Fig; S1 Table). Most traps operated autonomously using high-capacity batteries (27,000 mAh) allowing 3–4 days of function, or exceptionally powered by mains electricity at fixed or secured locations. Two attractants were used: an olfactory lure BG-Mozzibait – specifically designed to attract anthropophilic mosquito species by mimicking human skin scent– and CO_2_ from BG-CO_2_ Generators (Biogents, Germany), both effective for attracting Culicidae species [39,40].

**Fig 2:**
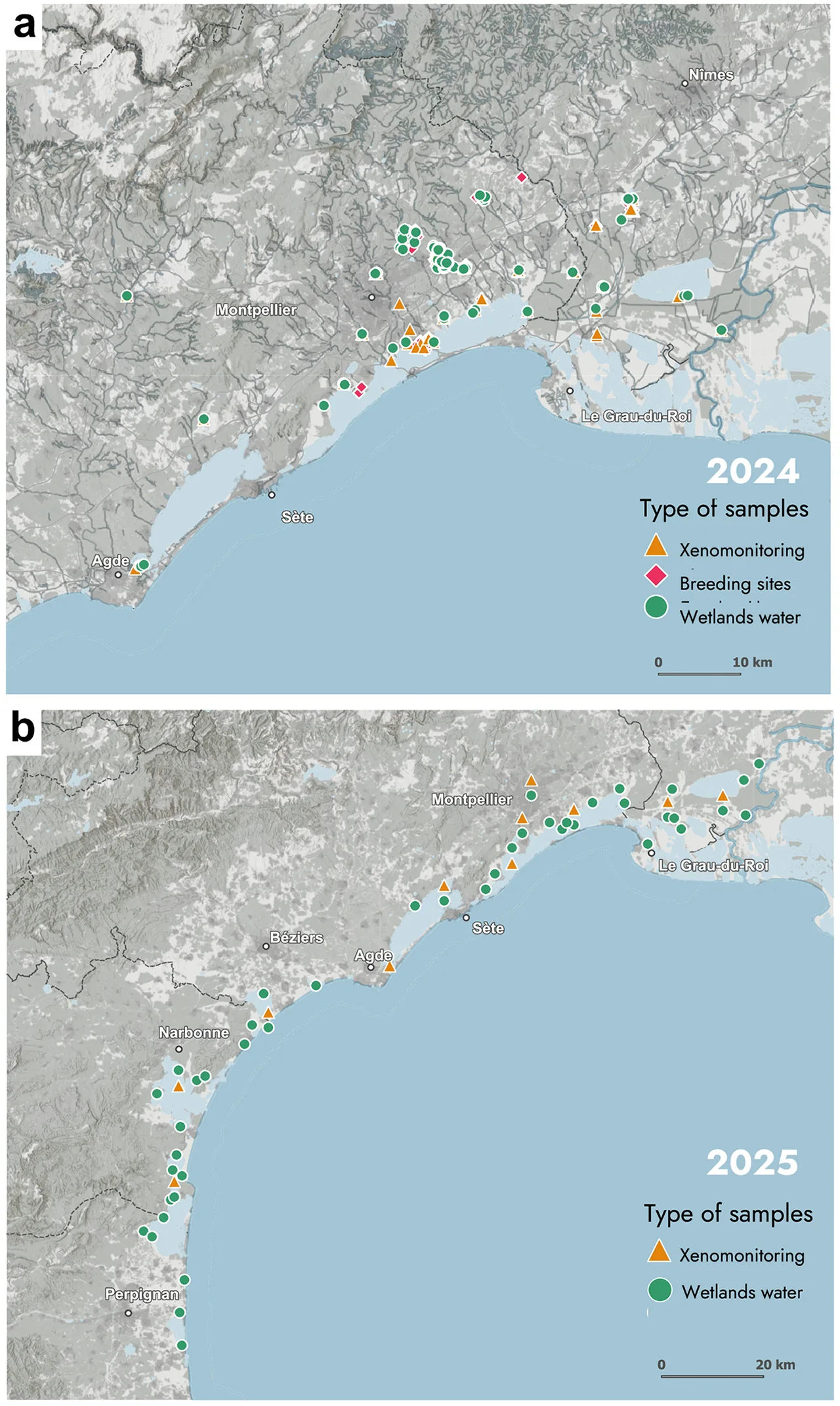
Location map of MX and water samples for 2024 (a) and 2025 (b) campaigns in Occitania, France. The basemap used for geographic visualization was derived from the *CORINE Land Cover (CLC)* dataset*,* provided through the *Copernicus Land Monitoring Service* of the *European Environment Agency (EEA).* https://land.copernicus.eu/en/products/corine-land-cover/clc2018. Licensing and terms of use are available at: https://land.copernicus.eu/en/faq, compatible with a CC BY 4.0 license. All spatial layers and overlays were generated and plotted by the authors using geographic information system (GIS) tools in QGIS.

In addition to routine surveillance, 27 MX traps were opportunistically deployed in 9 municipalities during vector control operations, following the same standardized protocol, baiting system and adapters, with traps usually active for 3–4 days.

Water collections in repeatedly sampled wetlands were conducted between 29 April and 8 October 2024, at 22 spots, and in 14 additional wetlands during vector control operations. Waters from artificial larval habitats were collected between 5 July and 14 October 2024, in 10 different municipalities harbouring a confirmed autochthonous human case with confirmed local arboviral circulation.

#### 2025 practical application to regional monitoring.

A sampling network of 45 wetlands and 11 mosquito traps equipped with MX adapters was deployed across Occitania, covering approximately 200km of the Mediterranean coastline, at fixed locations (Fig 2B; S2B Fig; S1 Table), from 1 April to 23 October 2025.

The wetlands, distributed across 29 municipalities, presented various hydrologic regimes (permanent, temporary or regulated), management intensities (from “natural” systems to heavily managed sites) and salinity level (freshwater, brackish or saline), but met the 2024 site types of interest: avian biodiversity hotspots, nesting and migratory stopover zones for wild birds, favourable habitats for *Culex* mosquitoes.

The 11 mosquito traps with MX adapters were systematically powered by mains electricity enabling continuous functioning. For sites open to the public, the traps were set in metal enclosure BG-Urban Trap stations (Biogents, Germany). The attractant system was set up to specifically target *Culex*: 10L pressurized cylinders released 0.5 kg/day of CO_2_ between 6:45 and 8:00 and between 19:00 and 00:30 [41].

MX adapters and water samples were collected biweekly. To best reflect the reactive and operational nature of this surveillance methodology, field sampling, trap collection, transportation, molecular analyses, and data interpretation were completed within one week.

For both 2024 and 2025 campaigns, all appropriate permissions and authorizations for trap setup and water sampling were obtained from relevant authorities or landowners. As mandated vector control operator for the Regional Health Agency of Occitania, Altopictus has access to confidential information regarding autochthonous human cases of WNV through the SI-LAV platform [30], including the date of symptom onset and the date of notification. These epidemiological data can be reported without compromising patient confidentiality. However, because of the inaccessibility of exact localisation of human or equine infectious transmission site, we compared the date of human or equine case alert with our detections at the departmental scale – the relevant scale to trigger an operational response following viral detection.

### Environmental water and MX analyses

#### Nucleic acid extraction from MX and environmental water.

Nucleic acid extraction protocols were optimized for both MX and water sample to maximize viral RNA recovery while maintaining compatibility with routine surveillance workflows. For MX samples, an extraction protocol (adapted from [23]) was implemented, in which aluminium foil collectors containing mosquito excreta were incubated in 1.2 mL of VXL lysis buffer (Cat. No.1069974, Qiagen, Germany) at room temperature (20–25°C) and vortexed for 5 min to improve solubilisation before automated nucleic acid extraction using Indimag Pathogen Kit (Cat. No. SP947457, Indical bioscience, Germany). The optimized workflow showed improved recovery compared with the initial in-house protocol and was subsequently applied to all MX samples from mid-2024 throughout the 2025 monitoring campaign (Detailed descriptions of extraction procedures and optimization experiments provided in S1 Text).

For environmental water samples, total nucleic acids were extracted using a protocol derived from a patented workflow [42] initially developed for pathogen detection in wastewater. The protocol was adapted to samples by including a pre-treatment step aimed at improving the recovery of viral particles associated with suspended solids. Comparative tests were performed on spiked samples to confirm improved recovery. The optimized protocol was then used for all water samples collected during the study (S2 Text).

#### Multiplex digital PCR assay and sequencing information.

A multiplex assay was developed to simultaneously quantify WNV and USUV. The WNV digital PCR (dPCR) assay targeted the 5’-UTR region [43], whereas The USUV assay targeted the NS1 region [44]. Primers were re-designed to improve specificity using MUSCLE sequence alignments [45] and Primer3 on Primer3Plus plateform*. In silico* specificity and lineage coverage were evaluated using nucleotide BLAST [41] against the GenBank database [42], aligning over 980 WNV sequences to ensure theoretical detection of the major circulating strains, including Lineages 1 and 2.

In addition to the two viral targets, the multiplex included a multispecies mosquito marker (“pan-mosquito”) used as a process control and an exogenous RNA control consisting of a non-target synthetic random RNA spiked into the reaction. This random RNA, quantified using a dedicated specific primer-probe set included in the multiplex, serves as a non-inhibition control to confirm the absence of PCR impairment.

Experimental specificity was assessed by RT-dPCR amplification by using RNA extracted from WNV and USUV. Cross-reactivity between both targets was evaluated, together with potential amplification of other arboviruses including Dengue virus (serotypes 1–4), Chikungunya and Zika viruses. Additional viruses commonly monitored in environmental surveillance were also tested: Hepatitis A virus (HAV), Measles virus (Wild-type strain), Respiratory syncytial virus A and B (RSV-A and B), SARS-CoV-2, Influenza A virus, H5N1 avian influenza virus and Norovirus GI/GII.

Digital PCR analyses were performed using the QIAcuity platform, enabling absolute quantification of viral RNA without external calibration curves. Analytical performance of the assay, including limits of blank, detection, and quantification, as well as optimisation of template input volumes for different matrices, is described in detail in S3 Text.

To confirm the specificity of low-level detections, we sequenced selected positive samples (Detailed sequencing workflows and analysis procedures provided in S4 Text). Whole-genome sequencing was also performed on high-viral-load MX or mosquito samples to determine circulating viral lineage, while targeted Sanger sequencing was performed using the dPCR forward and reverse primers (S2 Table and S4 Text)to ensure that near-threshold dPCR signals represented true amplification of the target sequence rather than primer-dimers or non-specific background noise.

### Data processing

Raw data generated by the QIAcuity Software suite V3.1.0.0. platform were interpreted based on the performance of internal and external dPCR controls. Positive dPCR controls for WNV and USUV were included at both high and low concentrations (3xLoD), to verify assay sensitivity, while the inhibition control was added to all samples and run alone at 3xLoD. Negativity of extraction and analytical control was checked. When acceptance criteria were met and at least 20,000 valid partitions were obtained, we set fluorescence threshold using positive controls and transformed raw partition counts into numbers of copies per mL, adjusting for the acid nucleic extraction process. Samples showing inhibition were re-analysed with a 1:10 dilution.

Data related to sampling site location and viral detection are publicly available [46]. Environmental and mapping data were collected in the field using the “MerginMaps” mobile application v2025.8.0 [47] on smartphone and processed with QGIS v 3.34.10-Prizren [48]. All analyses were carried out in R v. 3.6.1 [49] using the packages *dplyr* [50], *lubridate* [51] and *rnaturalearth* [52] for the analyses and data manipulation, and *ggplot2* [53] for data visualisation. OpenAI’s ChatGPT (version GPT-4.0, accessed January 2026) was used for R code improvement.

## Results

### Development and characterization of a digital-PCR method targeting WNV and USUV

#### Development of a reliable multiplex assay.

The dPCR assays for WNV and USUV, consisting in two primers and a TaqMan probe, were initially validated in silico through sequence alignment and BLAST analysis to assess theoretical specificity and lineage coverage. Sequence alignments across 980 WNV genomes confirmed 100% query coverage and 100% sequence identity within the 5’-UTR target region, demonstrating high conservation of this specific region and ensuring robust detection of both major European-circulating Lineage 1 and Lineage 2 strains. No significant matches with non-target viruses were detected.

Designed oligonucleotids (S2 Table) amplified a fragment of 105 bp for WNV in the 5’UTR region and a fragment of 88 bp for USUV in the NS1 region (S1 Fig). Each detection system specifically amplified its respective positive control in simplex assays without generating any signal for non-target viruses. No cross-reactivity was observed neither between WNV and USUV nor with any other tested viruses. The multiplex assay, comprising WNV, USUV, pan-mosquito and the inhibition control, was considered validated as the dPCR quantification of each target in multiplex did not decrease by more than 10% compared to the corresponding simplex assay, confirming that simultaneous detection did not compromise assay sensitivity or accuracy.

Inhibition tests were conducted to optimize assay sensitivity by maximizing sample volume input in the dPCR reaction mix while avoiding matrix-induced PCR inhibition. For MX samples, the comparison was performed on USUV-spiked samples. A detectable signal was observed for all USUV concentrations and volumes tested, except for the 2 µL input which did not allow detection of the lowest USUV-spiking concentration (Fig 3A). Because no quantification decrease or signal suppression was observed at higher volumes, confirming that the mosquito excreta matrix is free of PCR inhibitors, the maximum input volume of 8 µL was selected to achieve the highest possible analytical sensitivity for low viral loads. Conversely, environmental water samples collected from larval habitats and wetlands were highly complex and often concentrated in co-extracted inhibitors (such as humic acids and organic matter). When testing these samples (Fig 3B), increasing the dPCR input volume from 4 µL to 8 µL did not affect the qualitative detection of the inhibition control, but resulted in a significant decrease in the final quantification of the endogenous pan-mosquito target for both water samples. This confirmed matrix-induced inhibition led to the selection of a conservative input volume of 4 µL for environmental water samples to guarantee accurate quantification without compromising the PCR reaction.

**Fig 3:**
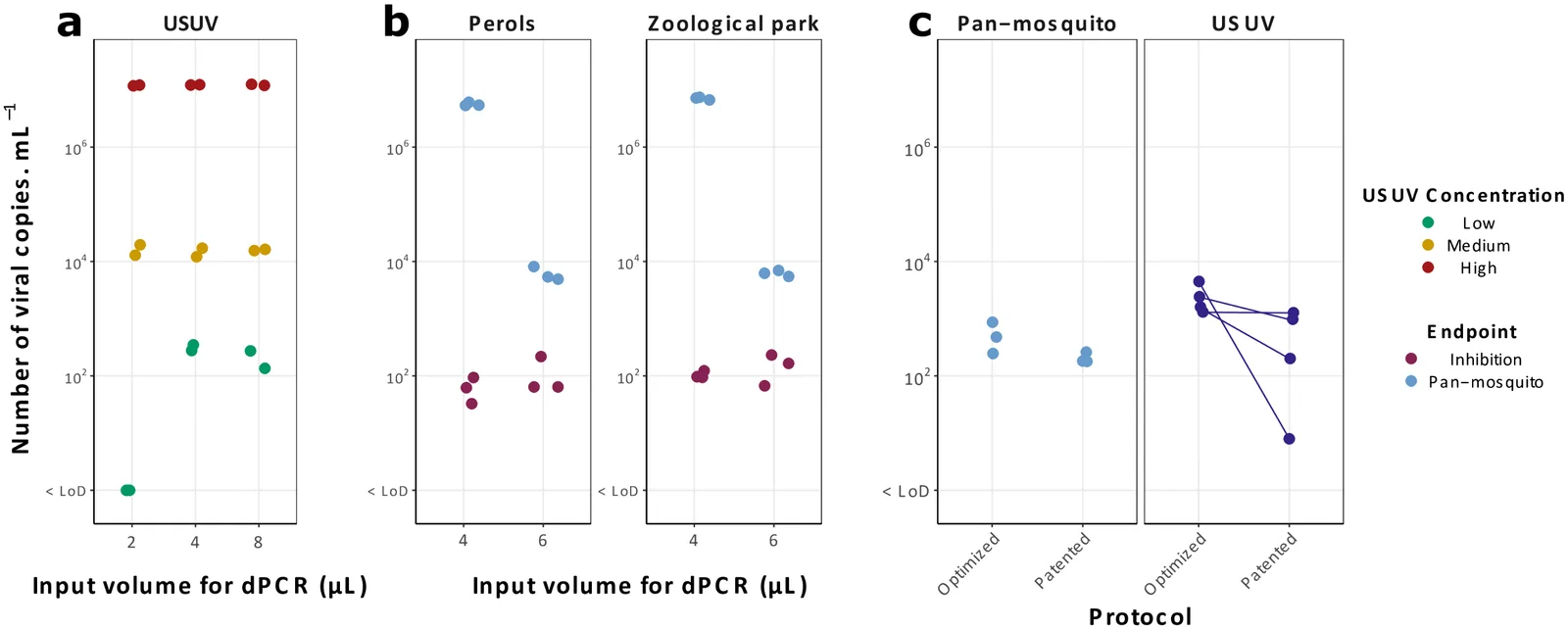
Digital-PCR and acid nucleic extraction methods optimization from mosquito excreta and water samples. dPCR inhibition assays were conducted both from **(a)** USUV-spiked MX samples, with 3 different sample input volumes (Mosquito excreta samples were spiked at Low: 4.6 TCID₅₀·mL ^-1^, Medium: 4.6 × 10^1^ TCID_50_·mL ^-1^, and High: 4.6 × 10^2^ TCID₅₀·mL ^-1^ concentrations), and (b) 2024 campaign artificial larval habitat samples (Pérols and Zoological Park), with 2 sample input volumes tested, for water analysis**. Water samples were not spiked, only pan-mosquito and inhibition control concentrations are shown. Extraction methods optimizations were conducted** in water samples, on (c) four USUV-spiked samples at a concentration of 1.10⁷ copies·L ^-1^ processed either with the patented or the optimized protocol, in addition to a non-spiked sample analysed in triplicates using both methods (only results for pan-mosquito target are shown). For all graphs, Y-axis is in logarithmic scale log10(x + 1). “<LoD” = below the limit of detection.

### Analytical performance

The analytical performance of the multiplex dPCR assay was measured by determining the LoB, absolute LoD, and absolute LoQ for WNV, USUV, and the pan-mosquito target (Table 1). LoB values were low for all detection systems, reflecting minimal background noise and allowing a clear distinction between true positive reactions and false-positive events. The LoB was slightly higher for the pan-mosquito target than for WNV and USUV but remained within an acceptable range for rare-target detection. The absolute LoD for both WNV and USUV was 0.125 copies per reaction, corresponding to the lowest concentration at which at least 90% of dPCR reactions yielded a positive signal. This demonstrates the high analytical sensitivity of the assay and its suitability for detecting low viral loads in environmental and MX samples. The absolute LoQ for WNV, USUV, and the pan-mosquito target was determined to be 1.25 copies per reaction. At this concentration, quantitative results met the predefined criteria for repeatability (RSDr ≤ 25%) and accuracy (±30% of the expected theoretical copy number). Compliance with the dMIQE 2020 guidelines [54] was ensured (S3 Table).

**Table 1:** Analytical performance of the WNV - USUV – Pan-mosquito multiplex assay. Limits are expressed in number of viral copies of target per µL dPCR reaction. LoD: limit of detection, LoQ: limit of quantification, LoB: limit of blank.

Target	Absolute LoD	Absolute LoQ	LoB
WNV	0.125	1.250	0.021
USUV	0.125	1.250	0.021
Pan-mosquito	0.125	1.250	0.030

### Development of an improved process for water nucleic acid extraction protocols

The optimized protocol resulted in higher detection levels for both USUV in spiked samples and the pan-mosquito target in larval habitat samples compared to the patented method across all tested conditions except one (Fig 3C). These results indicate an improved recovery and detection efficiency of viral and mosquito-derived nucleic acids using the optimized extraction workflow for environmental water matrices. The stability test performed on the water samples showed no decrease of WNV and pan-mosquito quantification after nucleic acid extraction up to 15 days post-collection (S4 Table), ensuring reliable storage for subsequent analysis. Consequently, the optimized protocol was selected and applied to all 2024 and 2025 water samples.

### WNV lineage identification classification and confirmation of dPCR assay specificity by sequencing

Over the two-year sampling period, seven specimens – two from MX samples, five from trapped mosquitoes– provided WNV viral loads high enough to enable whole-genome sequencing (Table 2). All sequences analysed belonged to lineage 2. In 2024, WNV-infected mosquitoes were detected in traps with negative MX samples but this discrepancy was no longer observed in 2025, likely due to the optimized MX screening protocol.

**Table 2:** Metadata for WNV sequences obtained from high positive samples in 2024 and 2025. Matrices were excreta collected by xenomonitoring, water from wetland or artificial larval habitats, or entire trapped mosquito individuals. *ND* stands for “Not Determined”, *NA* for “Not applicable”. The numbers next to the municipality names refer to S1 Table. Refer to S5 Table for Sanger sequencing.

Accession number	Sampling date	Matrix	Site type	Municipality	Lineage	Sequencing method	Genome coverage (%)	Sequence identity (%)
OZ263592	30/09/2024	Mosquito excreta	Natural reserve	Mireval (21)	2	WGS	51,11	*NA*
OZ263593	17/09/2024	Mosquito	Zoological park	Montpellier (23)	2	WGS	84,64	*NA*
OZ263594	13/09/2024	Mosquito	Marsh	Mauguio (19)	2	WGS	93,6	*NA*
OZ263595	16/08/2024	Mosquito	Natural reserve	Mireval (21)	2	WGS	93,6	*NA*
OZ263596	30/08/2024	Mosquito	Marsh	Saint-Laurent-d'Aigouze (4)	2	WGS	93,6	*NA*
OZ412497	29/07/2025	Mosquito excreta	Zoological park	Montpellier (73)	2	WGS	75	*NA*
OZ412496	09/09/2025	Mosquito	Educational farm	Saint-Jean-de-Védas (78)	2	WGS	94	*NA*
PZ530941	09/09/2024	Water (Artificial site)	Residential area	Baillargues (10)	*ND*	Sanger	*NA*	98,4
PZ530943	09/04/2025	Water (Wetland)	Saltmarsh	La Palme (37)	*ND*	Sanger	*NA*	98,5
PZ530944	09/04/2025	Water (Wetland)	Marsh	Narbonne (44)	*ND*	Sanger	*NA*	100
PZ530942	09/04/2025	Water (Wetland)	Temporary brackish lake	Fleury (34)	*ND*	Sanger	*NA*	96,9

Sanger sequencing was performed on selected WNV-positive samples using the dPCR primers to provide a qualitative confirmation of the dPCR signal. While sequencing such short fragment sizes (less than 105 bp) is unsuitable for phylogenetic analysis or lineage determination, it served as an independent double-control to definitively confirm sample positivity and rule out primer-dimers or artifacts in complex environmental matrices. The specificity of the underlying dPCR design was independently guaranteed by mandatory in silico screening—confirming the detection of both WNV Lineage 1 and Lineage 2—and extensive in vitro cross-testing using gBlocks against a broad panel of viral targets (including USUV, Dengue, Chikungunya, Zika, and RSV-A/B), which showed no cross-reactivity. No classical nested RT-PCR was performed on these samples due to the high risk of cross-contamination in routine surveillance and the lower sensitivity of nested primers on highly degraded environmental matrices. The short consensus sequences obtained from the samples aligned with West Nile virus reference genomes, confirming the amplification of the specific WNV target region.

All negative controls remained free of amplification, confirming the absence of contamination. For four low-positive samples (one artificial larval habitat from 2024 and three wetlands from 2025; ≤ 1 copy·µL^-1^ reaction), sequencing also produced high-quality reads aligning to WNV. Although the amplified region is known to share partial homology with USUV, the most divergent segment between the two genomes (positions 20–50 bp and 60–100 bp within the amplicon) was fully resolved. In both regions, nucleotide identity was consistent with WNV and not with USUV, ruling out a cross-amplification. Additional verification confirmed the absence of homology between the amplicon and the *Culex pipiens* genome, in agreement with the lack of reported viral integrations in this species. Altogether, these results demonstrate that the dPCR assay specifically detects WNV RNA, even at very low target concentrations, and does not produce nonspecific amplification from related viruses or host genomic material.

### 2024 operational proof of concept

In total, 508 water habitats were sampled, and 302 MX traps installed in 2024. WNV was detected 29 time in mosquito excreta and waters, in 11 different municipalities. From the repeatedly sampled sites, 9 out of 139 MX traps and in 8 out of 171 wetland water samples were positive, providing respectively up to 2.2.10^5^ and 9.8.10^2^ copies per mL (Figs 4 and 5). It was also detected during vector control operations in 1 out of 15 water samples from surrounding wetlands, 1 out of 32 MX traps and in 10 out of 45 artificial larval habitats.

**Fig 4:**
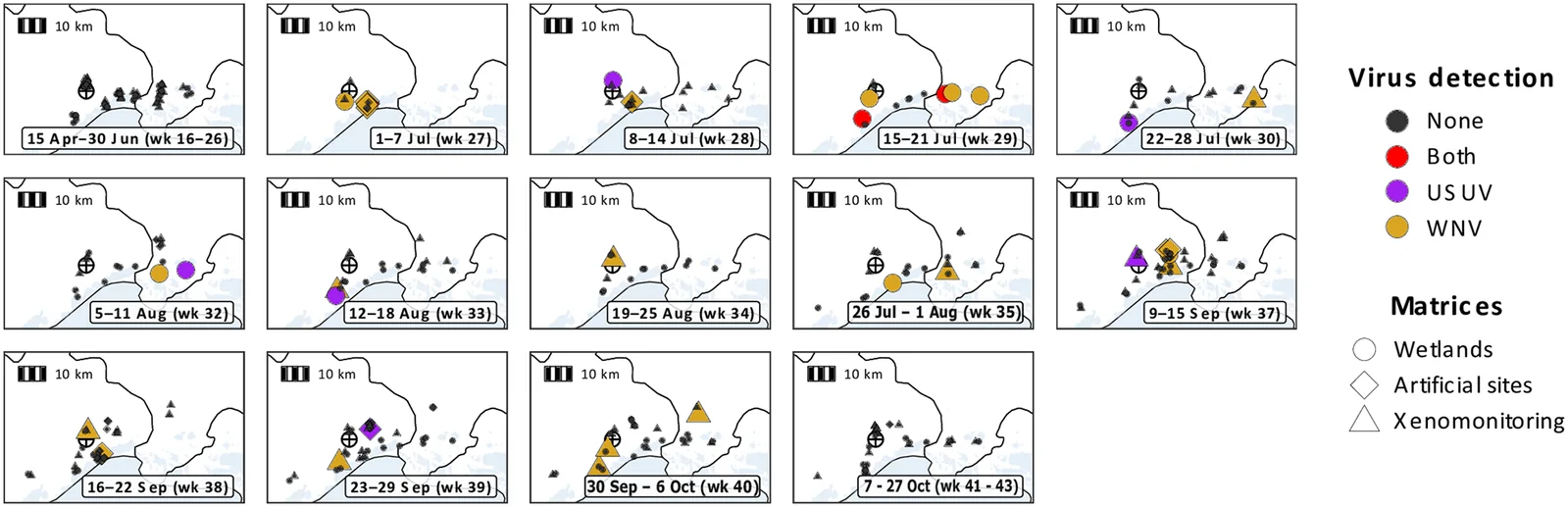
Maps of viral detection over the sampling season 2024 in wetland waters, mosquito excreta in MX adapters through xenomonitoring, or artificial larval habitats. The circled plus (⊕) symbolises the city of Montpellier. Overlapping points slightly jitter for graphical purposes. The basemap used for geographic visualization was derived from the *CORINE Land Cover (CLC)* dataset*,* provided through the *Copernicus Land Monitoring Service* of the *European Environment Agency (EEA).* https://land.copernicus.eu/en/products/corine-land-cover/clc2018. Licensing and terms of use are available at: https://land.copernicus.eu/en/faq, compatible with a CC BY 4.0 license. All spatial layers and overlays were generated and plotted by the authors using geographic information system (GIS) tools in QGIS.

**Fig 5:**
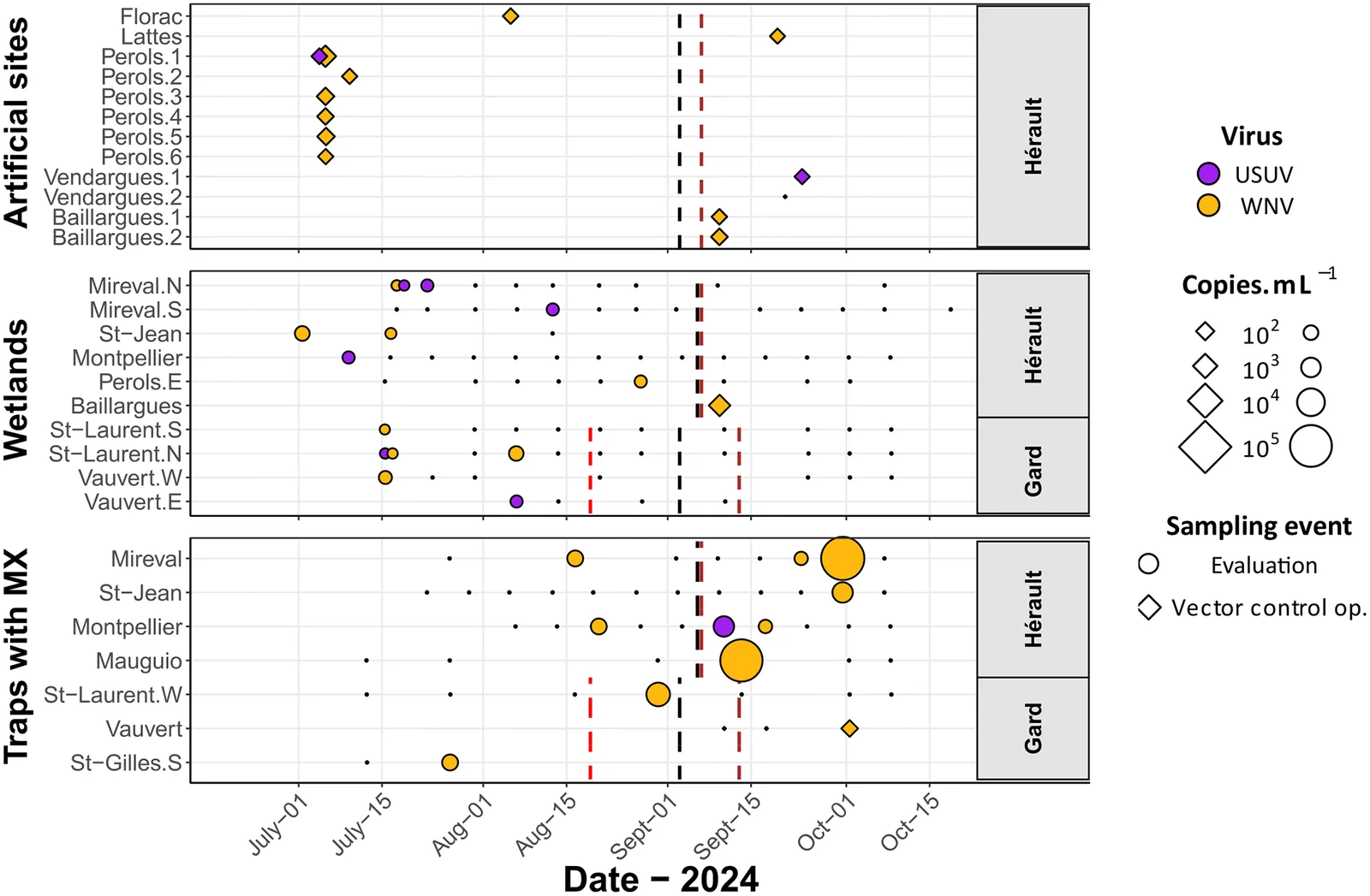
Number of viral copies of WNV and USUV per mL in artificial larval development sites (upper panel), wetland waters (middle panel) and in mosquito excreta collected in traps equipped with MX adapters (lower panel), from West (Mireval/Florac, top) to East (Baillargues, Vauvert and St-Gilles, bottom) in Hérault and Gard departments, regularly sampled from evaluation campaign or opportunistically collected during vector control operations, over the 2024 sampling season. The dashed lines indicate the first human detection of WNV in blood donations (in red), the first alert of human autochthonous case of WNV (in black) and the first equine alert of WNV (in brown) in their respective French departments in 2024. Small black dots represent sampling events with no WNV or USUV detection. Only sites with at least one positivity of either of the viruses are shown. Results for all sites displayed in S3 Fig. Overlapping symbols of simultaneous WNV and USUV detections slightly jittered for graphical purpose.

The earliest WNV detection happened on 1 July 2024 in a mixture of water from wetlands and poultry ponds in the educational farm of Saint-Jean-de-Vedas (Hérault). From the repeatedly sampled sites, WNV was last detected 30 September at the same location and in wetlands samples from the Natural Reserve in Mireval (Hérault). One MX sample was also WNV positive 1 October, during a vector control operation around the city of Vauvert (Gard). Comparatively, the first detection in human blood was 9 August in Gard [35], the first alerts of WNV autochthonous human case were 3 and 24 September for Hérault and Gard [30], and the first equine alerts of WNV, 6 and 13 September respectively [31,55].

USUV was detected nine time in six municipalities. It was present in one MX trap –3.2.10^3^ copies per mL– and in six wetland water samples –up to 62.5 copies per mL– from the repeatedly sampled sites, with a first detection 9 July at the Zoological park of Montpellier (Figs 4 and 5). Two artificial larval habitats from vector control operations were also positive, including the earliest detection 5 July in Pérols.

WNV and USUV were simultaneously detected in one artificial larval habitat during vector control operation in Pérols 5 July, in the Petite Camargue wetlands (Vauvert municipality) 15 July, and in the wetlands of the Natural Reserve of Mireval 17July.

### 2025 real-life campaign of regional monitoring

WNV was detected 76 times out of 705 water samples over the 28-week study period, collected from 40 distinct sites between 9 April and 23 October (Figs 6 and 7). Detection was the highest at the beginning of the campaign, with 32 and 26 WNV-positive water samples in April and May respectively, reaching concentrations of up to 1,984 copies·mL^-1^—the highest levels observed in this matrix. From June, the number of positive water samples declined, ranging from two to seven detections per month until October.

**Fig 6:**
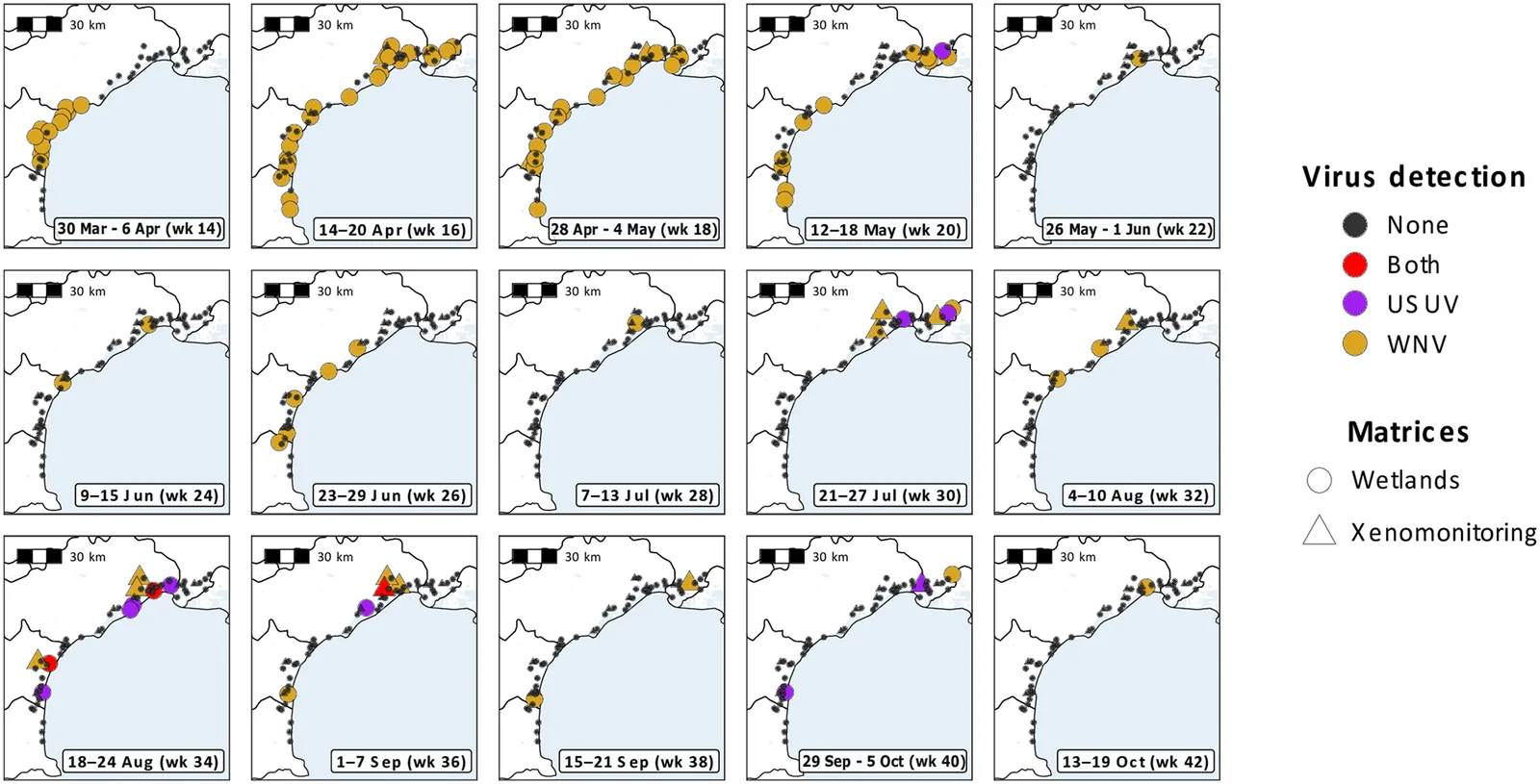
Maps of viral detection over the sampling season 2025 in wetland waters or mosquito excreta in MX adapters through xenomonitoring. The basemap used for geographic visualization was derived from the *CORINE Land Cover (CLC)* dataset*,* provided through the *Copernicus Land Monitoring Service* of the *European Environment Agency (EEA).* https://land.copernicus.eu/en/products/corine-land-cover/clc2018. Licensing and terms of use are available at: https://land.copernicus.eu/en/faq, compatible with a CC BY 4.0 license. All spatial layers and overlays were generated and plotted by the authors using geographic information system (GIS) tools in QGIS.

**Fig 7:**
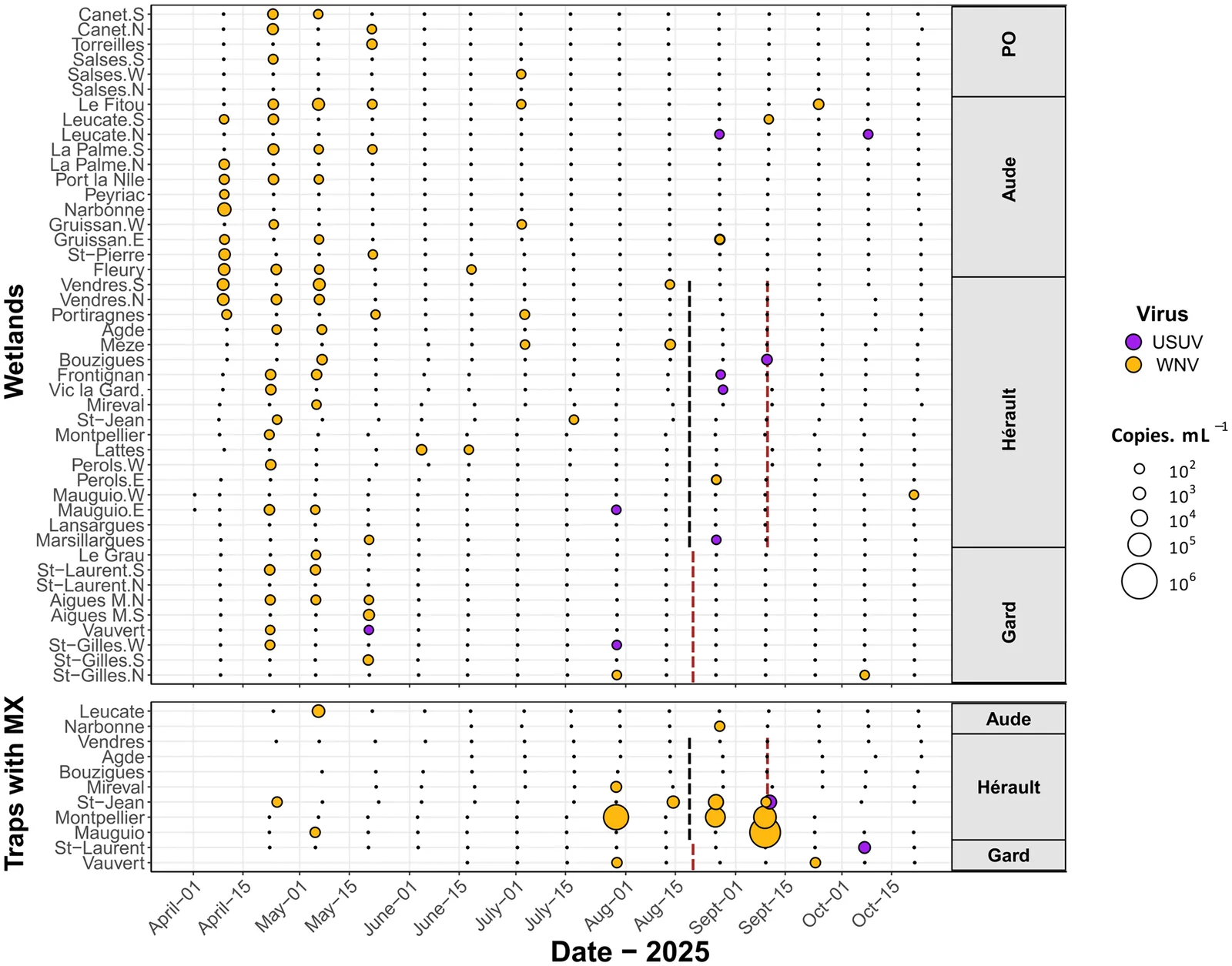
Number of viral copies per mL of WNV and USUV in wetland waters (upper panel), and in mosquito excreta collected in traps equipped with MX adapters (lower panel), from South-West (Canet.S and Leucate, top) to North-East (St-Gilles.N and Vauvert, bottom) in the Gard, Hérault, Aude and Pyrénées-Orientales (PO) departements, over the 2025 sampling season. The dashed lines indicate the first alert of human autochthonous case of WNV (in black) and the first equine alerts of WNV (in brown) in their respective French departments (where applicable) in 2025. Small black dots represent sampling events with no WNV or USUV detection.

Additionally, WNV RNA was detected in mosquito excreta in MX adapters at seven sites, with 14 detections out of 150 samples identified between 24 April to 23 September (Figs 6 and 7). Only one and two samples were positive in April and May respectively, three in July with up to 1.6.10^5^ viral copies per mL, and peaked in August and September with four positive samples each, yielding concentrations up to 3.9.10^4^ and 4.9.10^5^ copies per mL. Interestingly, WNV was detected both years at the educational farm of St-Jean-de-Vedas, the Zoological Park of Montpellier, and the Natural Reserves of Mireval (Hérault) and Vauvert (Gard). Comparatively, the first detection in human blood in the region was 25 August, the first alert of WNV autochthonous human case was the 11 August in Hérault [30]–while a first case showed symptoms the 18 July [56] but was recorded only the 21 August [30]–, and the first equine alerts of WNV, 20 August in Gard and 10 September in Hérault [31,55]. No such alert occurred in Aude and Pyrénées-Orientales departments.

USUV was detected 11 times in water samples from 10 sites, between 20 May and 8 October: once in May, twice in July, peaked in August with 6 positive samples then decreased the following months (Figs 6 and 7). USUV was also found twice in mosquito excreta in late season, 9 September in the educational farm, and in the Petite Camargue wetlands of the Natural Reserve of Vauvert (as in 2024) 7 October. Up to a 182 copies per mL were found in waters, but excreta yielded a maximum of ca. 3000 copies per mL, in September.

## Discussion

We developed an integrated surveillance strategy combining MX with water sampling from wetlands and mosquito larval habitats, supported by an innovative dPCR assay, enabling early and repeated viral detection during the 2024 and 2025 transmission seasons in Southern France. This multi-matrix approach detected WNV and USUV respectively 29 and 8 times in 2024, and 90 and 14 times in 2025, across 22 municipalities. Both years, WNV was identified weeks before autochthonous human cases or equine alerts in the region, highlighting a remarkable temporal precedence decisive for efficient arbovirus surveillance.

The detection of WNV and USUV in environmental waters required substantial methodological developments to ensure analytical specificity, sensitivity, and interpretability of low-level viral signals. The multiplex digital PCR assay targeting the WNV 5′-UTR and the USUV NS1 regions maximized specificity, with no cross-reactivity between WNV, USUV and a broad panel of non-target viruses. The characterization of the limits of blank (LoB), detection (LoD) and quantification (LoQ) proved the robust discrimination between true positives and background noises. Reported LoD for RT-qPCR assays optimized for WNV ranged from ca. 1.5 to 15 copies per reaction [57], and 8.8 copies per reaction [58] for multiplex RT-qPCR developed for environmental surveillance. For USUV, published RT-qPCR assays report LoDs around 50 copies per reaction in clinical matrices [59], and 11 copies per reaction in mosquito pools in optimized surveillance assays [60]. Despite the absence of LoB or LoQ, these studies demonstrate the high sensitivity and the reliable detection of qPCR assays at low viral loads, and provide relevant benchmarks for evaluating assay performance. In comparison, our dPCR system achieved a LoD of 0.125 copies per reaction for both viruses, reflecting the inherent advantage of dPCR in absolute quantification and partition-based detection. These improved quantitative precision and sensitivity are particularly relevant for environmental surveillance and samples with low RNA concentrations. This detection was further supported by qualitative Sanger sequencing of the short amplicons. Although these short fragments (less than 105 bp) do not allow for phylogenetic lineage characterization, they provided a valuable independent double-control to confirm WNV target amplification.

The optimized water workflow improved the recovery of insoluble fractions in environmental waters, where enveloped viruses may associate with suspended solids [61], although the extraction efficiency may vary with environmental conditions. Matrix heterogeneity and the presence of amplifications inhibitors represented important practical constraints. Increasing input volume improved representativeness for mosquito excreta without measurable inhibition, whereas water samples required smaller inputs to accommodate heterogenous inhibitor contents across wetlands and artificial larval habitat waters. Less sensitive to partial inhibition than qPCR–widely used in arboviruses surveillance [62,63]–, dPCR allows absolute quantification without external standard curves but still requires matrix-specific optimisations [64,65]. Additionally, some robustness and repeatability parameters recommended by the dMIQE guidelines [54], originally defined for homogenous standardized clinical samples, could not be fully addressed, since environmental matrices show strongly heterogenous physicochemical properties across sites. We therefore prioritized the selection of a versatile protocol applicable across diverse environmental matrices, and the workflow proved robust and operationally applicable across highly heterogenous matrices. The superior sensitivity and tolerance to inhibitors of digital PCR contributed to the early viral detection in environmental and mosquito-derived matrices, illustrated by samples positive by dPCR but undetectable by qPCR. The discrepancy observed in 2024, where WNV-positive mosquito pools were occasionally associated with negative MX samples, likely reflects the lower recovery efficiency of the initial MX extraction workflow rather than a biological inconsistency. Because viral RNA concentrations in mosquito excreta are expected to be substantially lower than in infected mosquito tissues, incomplete recovery during the extraction step may have resulted in false-negative MX samples. The improved excreta solubilization and nucleic acid recovery implemented in the optimized protocol likely increased analytical sensitivity, providing a plausible explanation for the absence of this discrepancy during the 2025 surveillance campaign. Future molecular improvements (e.g., targeted enrichment strategies, hybridization-based capture methods, optimized multiplex amplification) could enhance the sequencing potential of environmental waters, to recover longer genomic fragments and enabling confirmation of early detections and genomic characterization of circulating WNV and USUV lineages.

The integrated use of MX and environmental water sampling provided a marked temporal advantage over conventional alert and associated surveillance systems. In 2024, at the departmental scale, WNV was identified in wetland water samples up to 60 days before the first alleged equine case in Gard [35], 60 days before the first equine alert [31] in Gard and 67 days in Hérault, 35 days before the first detection in human blood in Gard [35], and 50 days before the notification of the first autochthonous human case in Gard and 67 days in Hérault [66]. In 2025, at the same departmental scale, the detection in water preceded the first alert of an autochthonous human case up to by 33 days in Hérault with no corresponding alert in the other departments. The precocity of detection was even greater in equine cases, with the first water detection preceding the first case by 9 weeks in Hérault, and by 17 weeks in Gard; no alert was issued in Aude and Pyrénées-Orientales. Such early detection and the absence of human or equine alerts in some departments underscore the capacity of environmental matrices to capture viral circulation before clinical or veterinary reported –if reported at all– cases, a critical asset given the high proportion of asymptomatic WNV and USUV infections.

Detection of WNV in water or mosquito excreta provides evidence of viral circulation in the environment, which may or may not coincide with human or equine cases, whether due to genuine absence of transmission to human or equine compartment, or a failure to detect infection (e.g., asymptomatic or unreported cases, misdiagnosis). Conversely, human or equine cases may occur in areas where environmental surveillance did not detect the virus. The probability of detecting WNV in water or mosquito excreta is likely influenced by several factors affecting viral input and amplification, including the abundance, community composition and infection status of avian hosts [67], as well as mosquito abundance and infection prevalence [68]. Viral persistence and detectability in environmental matrices may additionally be affected by physicochemical conditions such as temperature and solar/UV radiation [69], and finally, by the probability of sampling viral materials (e.g., the distance between infectious birds and the sampling site). These limits, inherent to any surveillance systems, underscore the needs of complementary approaches.

Nevertheless, our results demonstrate that the method is operationally sound, effective, capable of providing early detection of WNV circulation, compared to other methodologies. For instance, surveillance in resident and migratory birds [70] also enables early detections–as early as March [16,71]– but requires substantial logistical investments. In comparison, water analysis detected the viruses from the beginning of the sampling campaign in April, indicating that our methodology may provide detection within comparable timeframes. However, for both target viruses when viral materials were detected in both matrices, detections in environmental water but one occurred weeks before detection in mosquito excreta within the same geographic area (Fig 8). Such early detections likely reflect the capacity of the aquatic environments to integrate multiple potential viral sources, such as excretion [28] and decay [72] of birds, avian shedding [73], or mosquito excreta [27], functioning as an early indicator of local viral circulation. Environmental detection of WNV and USUV may provide evidence of their concomitant circulation [74] and may also reflect potential interactions in their transmission dynamics [75]. Water-based screening can facilitates early localisation of transmission hotspots, while concomitant MX monitoring strengthens inference on vector involvement and enables viral sequencing and lineage identification for expanded genomic surveillance [36,76].

**Fig 8:**
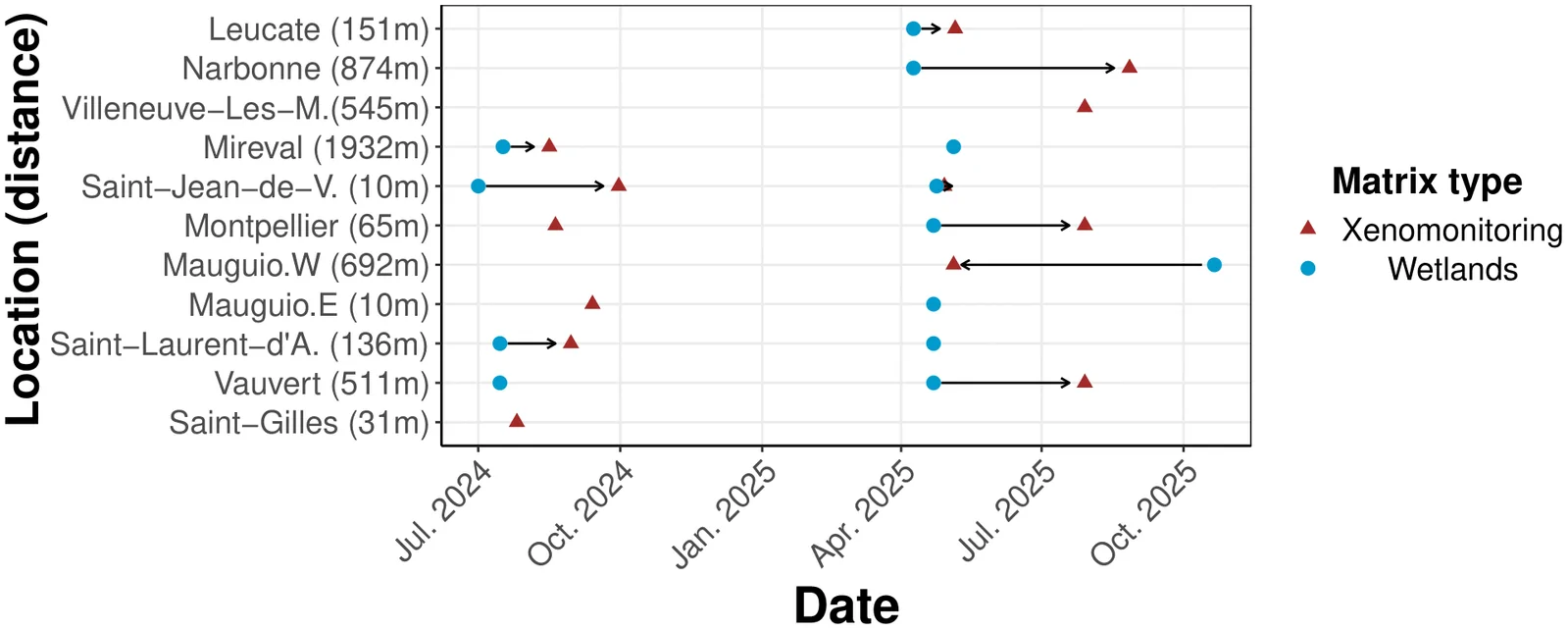
Dates of earliest WNV detection in Xenomonitoring, and in wetlands water from the nearest sampling point from the trap (distance indicated next to the location name, in meter) in 2024 and 2025. Arrows point from detection in wetland water to detection by xenomonitoring.

Low-level viral RNA detection in environmental matrices is not a direct evidence of ongoing transmission but, when supported by a validated analytical framework, repeated samplings and/or orthogonal confirmation, this framework provides a robust basis for proactive surveillance and public health actionable risk signals. Beyond analytical sensitivity, the primary contribution of this multi-matrix strategy is its decision-support value at the territorial scale. Since human and veterinary surveillance systems are intrinsically delayed, early detection in environmental waters and mosquito excreta could trigger time-sensitive actions, including vector control operations, communication to public and health professionals, intensified One-Health surveillance, and preparedness to arboviral expansion [77]. *Culex* population controls remain essential for reducing WNV and USUV transmission. Their long-distance dispersal, short adult lifespan, and the dead-end role of humans and horses, limit the relevance of adulticide measures [78]. Control strategies therefore relies on large-scale, regular larval habitat treatments, requiring early and accurate identification of viral circulation areas to optimize resource allocation [79]. Our simplified yet diversified surveillance strategy, covering broad geographic areas throughout the vector season, can enable such timely early warning signals and localisation to guide targeted interventions, avoiding more costly, untargeted extensive larvicide treatments. The detection of WNV in artificial larval habitats around autochthonous human cases validated its capacity to identify ongoing local viral circulation, to guide urban vector control operation, and to describe spatial and temporal co-circulations of WNV, USUV, and *Aedes*-borne viruses within shared environments.

Besides the expected advantages of the method, several limitations should be acknowledged, while also providing avenues for further improvement. Analytically, environmental matrices show substantial physicochemical heterogeneity (such as salinity, organic load, hydrological regime) across sites, which constrains the standardisation of extraction protocols and prevents full compliance with dMIQE robustness and repeatability recommendations designed for homogeneous clinical samples [54]. The short amplicon size (<105 bp) required for reliable amplification also precluded phylogenetic lineage characterisation directly from water. Operationally, the 2024 and 2025 campaigns differed in design: sampling in 2024 was opportunistic and partly associated with vector-control operations, whereas the 2025 campaign followed a systematic, fixed-site design with mains-powered equipment and biweekly sampling. This difference limits direct year-to-year comparison of detection rates although the standardised design of the 2025 campaign improved the consistency and reliability of the dataset. Nevertheless, both campaign highlighted the importance of careful site selection to maximise detection probability—considering factors such as avian abundance and community composition, water and wind flows, environmental conditions favourable to both birds and mosquitoes—while also facilitating field operations –site accessibility, water permanence, optimised sampling routes. Finally, establishing causal relationships between environmental viral detections, spillover into human or animal populations, and epidemiological parameters known to influence arbovirus transmission was beyond the scope of the present study. Addressing these relationships should however be a priority for future research to assess the added value of our methodology to current surveillance systems. An important challenge for fine-scale spatial analyses will be the confidentiality of human and equine case locations, which prevents the disclosure of their precise geographic coordinates.

In conclusion, our approach offers a scalable, field-compatible framework to support territorial decision-making (Fig 9) by enabling earlier targeting of surveillance and vector control resources. In practice, we propose an operational tiered interpretation of signals: (i) a single low-level detection above the LoB/LoD prompts confirmatory re-sampling and local risk assessment, (ii) repeated detections or signals across multiple matrices trigger geographically targeted prevention measures, and (iii) sustained signals across weeks support escalation of territorial preparedness and integrated One Health surveillance. The limitation of the largely reactive and weakly predictive traditional systems are illustrated by the costs [80,81] and the low positive sample ratios [82,83] in the well-structured WNV surveillance programs in northern Italy. At that point of development, our methodology still requires formal quantitative assessments of its cost-effectiveness –in terms of detection timeliness, financial costs, human resources, logistical requirements, and operational workload– compared with conventional surveillance systems, to determine the conditions under which this approach provides a meaningful operational benefit and to support its integration into routine surveillance strategies. Nevertheless, by providing a sensitive, proactive framework for early detection of emerging *Culex*-borne arboviruses, environmental multi-compartment surveillance, alongside predictive modelling and established One Health networks, has the potential to offer substantial lead time to guide public health interventions and to support more anticipatory risk assessment and response capacity in endemic and emerging regions.

**Fig 9:**
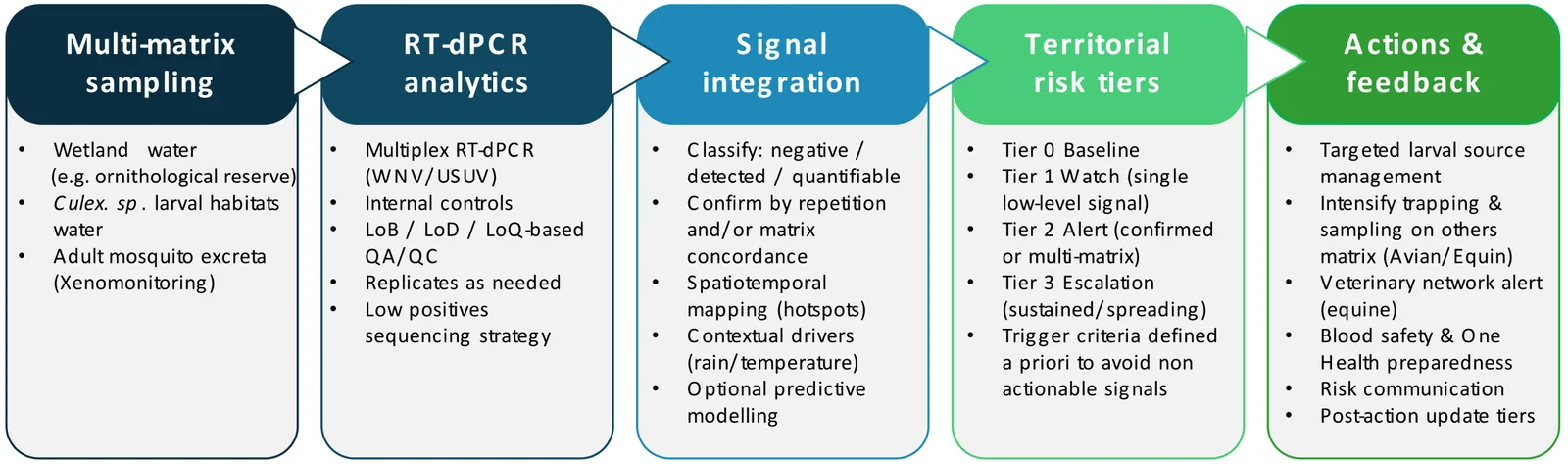
Territorial decision-support pipeline translating multi-matrix RT-dPCR surveillance signals into One Health actions.

## Supporting information

S1 Fig Example of positive and negative raw results for WNV (A) and USUV (B). Blue dots represent positive partitions, grey dots negative partitions. The red line represents the threshold separating positive and negative partitions.
(TIF)

S2 Fig Maps of sampling sites for 2024 (A) and 2025 (B) campaigns. Numbers refer to sampling sites described in S1 Table.
(TIFF)

S1 Table Sampling points for 2024 and 2025 campaigns. “USUV detection” and “WNV detection” indicate the detection of the virus at the sampling point at least once. The basemap used for geographic visualization was derived from the CORINE Land Cover (CLC) dataset, provided through the Copernicus Land Monitoring Service of the European Environment Agency (EEA). https://land.copernicus.eu/en/products/corine-land-cover/clc2018. Licensing and terms of use are available at: https://land.copernicus.eu/en/faq, compatible with a CC BY 4.0 license. All spatial layers and overlays were generated and plotted by the authors using geographic information system (GIS) tools in QGIS.
(XLSX)

S3 Fig Number of viral copies of WNV and USUV per mL in artificial breeding sites (upper panel), wetland waters (middle panel) and in mosquito excreta collected in traps equipped with MX adapters (lower panel), from West (Mireval/Florac, top) to East (Baillargues, Vauvert and St-Gilles, bottom) in Hérault and Gard departments, regularly sampled from evaluation campaign or opportunistically collected during vector control operations, over the 2024 sampling season. All sites displayed. The dashed lines indicate the first human detection of WNV in blood donations (in red), the first alert of human autochthonous case of WNV (in black) and the first equine alert of WNV (in brown) in their respective French departments in 2024. Small black dots represent sampling events with no WNV or USUV detection.
(TIF)

S2 Table Primers and probe targeting WNV and USUV viruses. (DOCX)

S3 Table dMIQE2020 checklist for authors, reviewers and editors. (XLSX)

S4 Table Stability test Pan-mosquito and WNV viruses over time. (DOCX)

S5 Table Sequencing by Sanger. (DOCX)

S1 Text Method development for nucleic acid extraction from MX samples. (DOCX)

S2 Text Method development for nucleic acid extraction from wetlands and artificial site water samples. (DOCX)

S3 Text Digital-PCR method characterization and quality control set-up. (DOCX)

S4 Text Virus sequencing. (DOCX)
