# Supplementary material for: Early-warning surveillance of West Nile and Usutu viruses in wetland and larval habitat waters and in mosquito excreta using digital PCR
Source: PLoS Negl Trop Dis. 2026 Oct 6;20(10):e0014799. doi: 10.1371/journal.pntd.0014799 (PMC13657283; doi:10.1371/journal.pntd.0014799)
Supplement: Supplementary file 1 [file pntd.0014799.s014.pdf]

We sincerely thank the Editor and Reviewers for their careful and constructive comments. We have revised the manuscript accordingly and have clarified the methodological limitations, funding information, sequencing results, figures, and interpretation of the findings. All changes are tracked in the revised manuscript.

(Note: the Line numbers refer to the lines in the clean, untracked document)

Reviewer #1:
Q3 : The authors provide some important information about habitats that is unfortunately, in general, missing from many published field experiment methodologies for vector surveillance. However, they have done this in a very vague way. How did they define any of these "epidemiologically relevant" parameters? How to identify an "avian biodiversity hotspot" (L118, L161) or "bird-dense" waters (Fig8)? For mosquito trapping, I think it is important to provide a minimum description of sampled habitats, but the authors should take care when using such vague terminology for specific parameters. They do not seem to be "predefined" (L139, were these the same criteria used to define environmental sampling?) They don't necessarily need to provide this to any great level of detail (site-specific parameters), as description of multiple sampling sites across a regional scale would be burdensome and would not add a lot to the manuscript. However, it is an important limitation of their ability to infer the predictive power of their surveillance schema. Therefore the authors should consider using less vague terminology, and should absolutely address the fact that their implementation experiment was not designed to show how useful this system can/could be, rather that it can be implemented on a wide geographic scale.

R3 : We agree with the reviewer on the importance of providing information on the habitats sampled, particularly for a system intended to be deployed at a broad geographic area. We have therefore added additional information to Supplementary Table S1, including vicinity type, water type for water samples, and comments describing the sampling sites. Ultimately, we intend to provide detailed site-specific parameters, but we cannot currently make all of these data publicly available because detailed land-cover and other site-specific environmental parameters will be used in a subsequent study. Following publication of that work, the Dryad dataset associated with the present study will be updated to include these additional variables while retaining the same DOI.

Regarding the terminology used to describe sites, some of the terms were indeed insufficiently specific. These terms were not intended to refer to formally defined or quantitatively established habitat categories. We used descriptive categories to identify sites considered relevant to the surveillance of mosquito-borne pathogens, based on the presence or expected concentration of key epidemiological components, including birds, vectors, and human or animal cases. In the revised manuscript, we have clarified these descriptions and added examples. For instance, an example of an “avian biodiversity hotspot and congregation site” has been provided (L127), such as an ornithological natural reserve. Similarly, the term “bird-dense” has been removed from Figure 8 and replaced by the more specific example “e.g., ornithological reserves”.

The reviewer is also correct that these categories were not based on strict predefined quantitative criteria. We have therefore replaced the sentence L169 with the more accurate wording: “Based on the aforementioned ecologically and epidemiologically relevant site types”.

The “Mosquito excreta” paragraph has been displaced L148, below the “Wetland and artificial breeding site water collection” paragraph for editorial reasons: it was easier to describe the localisations of the traps after the description of the wetlands sites.

We agree with the reviewer that the study was not designed to assess the predictive performance of the surveillance system, as would be required to determine its predictive power in a modelling framework. The objective of the present study was instead to demonstrate that the surveillance strategy could be implemented operationally across a wide geographic area, and that the information generated could be used to support or trigger operational decisions, like early control and prophylactic measures. Our study demonstrates the operational feasibility and geographic scalability of the surveillance system, assessing its predictive capacity and its ability to anticipate pathogen transmission or outbreak risk would require a dedicated study and is beyond the scope of the present implementation experiment.

Q4 : L143 - the authors must note that BG-Mozzibait is not intended for use with ornithophilic Culex species.

R4 : We have revised the description and added : « – specifically designed to attract anthropophilic mosquito species by mimicking human skin scent– » (L174) in the description of the Mozzibait

Reviewer #2:

Q5 : page 4 L. 63: Correct "to date, no human antiviral treatments or vaccines exist for both viruses."

R5 : The sentence L.65 was revised following the reviewer's recommendation

Q6 : page 5 L. 75-79: The paragraph presented 3 major ideas but they need to be more connected, linked to each other. Please rephrase this paragraph to linked it with surveillance improvment.

R6 : We have rewritten the paragraph L.74-85, keeping the two main issues (detection of low-level circulation and costs of current operational responses) and linking them to improvement surveillance.

Q7 : page 5 L. 88-91 : Add a reference explaining in what situation (of which virus) it has been a good way/method of surveillance ? (For which virus this technic has allowed an efficient surveillance /anticipation of the risk of emergence)

R7 : We have clarified that MX is an efficient method for real-time monitoring of WNV and USUV circulation, but that viral detection in mosquito excreta provides evidence of ongoing viral circulation rather than a predictive signal of virus emergence (L.89). We further added a reference showing that detection of viral RNA in wastewater can precede the detection of clinical cases and hospitalizations for SARS-CoV-2. Although such environmental surveillance may provide an early warning signal to support enhanced surveillance and control measures, the environmental detection of WNV or USUV has not yet been quantitatively linked to a subsequent outbreak.

Q8 : Page 7 L. 110-111 : I was not able to find those results dealing with the confirmation of presence of the virus in the Culex females using qRT-PCR ? In which tables those results are presented ? Those results deserve to be presented, as they will allow the confirmation of the sequencing results.

R8: We believe that the reviewer is referring to the qRT-PCR analysis performed on Culex females collected from traps associated with a positive mosquito-excreta (MX) detection (L.161).

We would like to clarify that viral RNA detection in mosquito excreta was performed by IAGE using RT-dPCR. Following a positive MX detection, the Culex females collected from the corresponding trap were pooled and subjected to an independent WNV/USUV duplex qRT-PCR analysis as a complementary entomological investigation. This qRT-PCR analysis was not performed by IAGE and was not part of the RT-dPCR analysis of the MX samples.

We agree that the wording in the Methods could have led to the misinterpretation that these qRT-PCR analyses constituted a confirmation of the sequencing results. We have therefore revised the relevant section to clearly distinguish the RT-dPCR detection performed on mosquito excreta from the complementary qRT-PCR analysis performed on mosquitoes collected from the corresponding traps.

Q9 : Page 7 L. 120: Correct “history of WNV/USUV circulation, sites with confirmed human or animal cases…”

R9 : The sentence L.128 was revised following the reviewer's recommendation

Q10 : Page 9 L. 157: Suppress “ca” after covering , replace by “approximately “ ?

R10 : Word replaced L.188

Q11 : Page 10 L. 179: Add the volume of VXL lysis buffer you use (even if this is presented in the supplemental data). Also add the information regarding at which temperature did you process your samples

R11 : We agree with the Reviewer that these parameters are important for reproducibility. We have updated the Methods section (L.222) to include the exact volume of VXL lysis buffer used (1.2 mL) as well as the incubation temperature (room temperature, 20–25°C).

Q12 : Page 11 L. 197: It is important to mention what “exogenous RNA inhibition control” you have use for your test. I was not able to find this information in the supplemental data neither

R12 : The exogenous RNA control consists of a non-target, synthetic random RNA sequence that has no homology with known viral or host genomes. This synthetic RNA is spiked into each reaction and quantified using a dedicated, highly specific primer-probe system. Successful amplification and quantification of this target serve as a "non-inhibition" control, confirming the absence of PCR inhibitors in the reaction. We have updated the Methods section to clarify this point (L. 243-246).

Results

Reviewer #1: **Q13** : L296, L400-401. Sanger sequencing an amplicon from a Taqman RT-qPCR assay is not a good laboratory practice, particularly in diagnostics. Their results (Supplemental table) are unconvincing, and, frankly, I cannot believe they submitted these to GenBank (as the database suffers from poor quality unverified sequences). I would remove these from the manuscript (and rescind the submission to the database, if possible). Aligning the sequences provided in the manuscript to publicly available sequences does not provide an "unambiguous" (L299) identification.

Reviewer #2: **Q20** : Table 2 : The size of the amplicon of sanger sequencing is really really small (less than 80nt), and from my point of view this is problematic for a confirmation step… Moreover I was not able to find the primers you use for this confirmation ? It seems it was the one of the dPCR ? From my point of view, when you want to confirm a new method of detection, you should confirm your results with other “recognized methods/published ones” like the WGS used for highly positive samples… For low positive samples, classical nested PCR should have been used, a pan flavivirus one, to be able to detect and confirm your findings… If this was done without success, it should be mentioned in the manuscript.

R13 and R20 : We fully agree that sequencing short fragments (<105 bp) directly derived from a dPCR TaqMan assay design is not standard diagnostic practice and is inherently unsuitable for robust phylogenetic analysis or lineage determination.

To clarify our workflow, the primary objective of Sanger-sequencing was not to perform a formal genetic or lineage characterization of these low-viral-load environmental samples. Instead, because this workflow describes the detection of WNV RNA in highly complex environmental matrices for the first time, this step was included as a planned, qualitative safeguard in our project. Its sole purpose was to confirm the positivity of our samples by acting as an independent double-control. Whole-genome sequencing was performed for robust lineage determination. Sanger-sequencing only ensured that near-threshold dPCR signals represented true amplification of the target sequence rather than primer-dimers or non-specific background noise.

Regarding the Reviewers' methodological queries:

1. **Primers used:** We confirm that the Sanger sequencing was performed using the dPCR primers themselves. This has now been explicitly stated in the Methods section of the revised manuscript. (L. 354)
2. **Nested PCR:** We did not perform classical nested RT-PCR or pan-flavivirus nested PCR on these specific samples. While nested PCR is a valuable tool for amplifying low-viral-load samples, it also carries a well-documented and high risk of laboratory cross-contamination in high-throughput surveillance settings. Additionally, multi-step nested PCR often exhibits lower sensitivity and reproducibility than optimized real-time dPCR assays when dealing with highly degraded RNA from environmental matrices (such as brackish runoff water).

Instead of relying on post-detection nested PCR confirmation, the absolute specificity of our assay was guaranteed *prior* to this study through a rigorous, multi-step pre-analytical and analytical validation protocol:

- **In silico validation:** Extensive alignment and BLAST analyses confirmed that the primers and probe set target a highly conserved region of the 5'-UTR, successfully recognizing both WNV Lineage 1 and Lineage 2 strains (L239).
- **In vitro specificity testing:** The assay was experimentally cross-tested using synthetic gBlocks and reference materials against a comprehensive panel of target and non-target pathogens, including WNV, USUV, Dengue virus (serotypes 1–4), Chikungunya virus, Zika virus, Respiratory Syncytial Virus (RSV-A and RSV-B), and other common environmental viral targets. No cross-reactivity or non-specific amplification was observed.

Therefore, while the Sanger amplicons are too short for phylogenetic assignment—which is why these samples are strictly designated as "ND" (Not Determined) for lineage in Table 2 (L.377)—the internal sequence data, acting as a qualitative double-control, formally confirms the positivity of our WNV detections.

We have revised the manuscript to transparently state these limitations (L.242-247 and L.349-375), mention the primers used (L. 354), and tone down the terminology (removing the word 'unambiguous'; L.363-365)

Reviewer #1:

Q14 : Figures 5 and 7: these dashed lines are suspicious, as they seem to represent very different geographic scales than the results of trapping - making this figure ambiguous, at best, and misleading, at worst. The authors have provided links for the data, but the links do not count as "publicly available" if they are require a login. Publicly available data from Santé publique France list Week 29 (July 14.-20.) as the first autochthonous human case of WNV in Occitanie (in Hérault) in 2025 - this differs from what is shown in the figure and in the text (L372).

R14 : The dashed lines delineate the administrative department, which is the lowest operational scale at which vector control and public health responses would be implemented following the detection of a human WNV case (e.g., currently, the blood donation surveillance). Accordingly, each dashed line encompasses all sampling sites located within the corresponding department. The department names were also added in the figure for clarity.

We also recalculate the delays between detection in water and equine/human cases at the departmental scale (L.445)

There was an inconsistency regarding the WNV-positive blood donation in 2024. This detection occurred in the **Gard** department only, and the dashed line has been corrected accordingly to refer only to that department.

The data from the SILAV platform is not publicly accessible because it contains confidential health information. However, as the vector control operator mandated by the Occitanie Regional Health Agency, Altopictus has access to these data and can report the dates of symptom onset and notification/alert without compromising patient confidentiality. To clarify this point, the following sentence has been added to the Materials and Methods section (L.208):

> As mandated vector control operator for the Regional Health Agency of Occitania, Altopictus has access to confidential information regarding autochthonous human cases of WNV through the SILAV platform[37], including the date of symptom onset and the date of notification. These epidemiological data can be reported without compromising patient confidentiality.

Indeed, as mention in the Santé Publique France’s bulletin, the first autochthonous human case of WNV showed first symptoms the 18th of July 2025. However, the corresponding alert only occurred the 21st of August. The first alert of the year in Occitania occurred the 19th of August, regarding another autochthonous case exhibiting symptoms the 9th of August. The discrepancy between the dates is now explained in the text (L.452), highlighting at the same time the lack of efficient surveillance of WNV. We also corrected the figure, as no detection occurred in blood donation in 2025, the date previously present in the figure constituted a detection following an organ transplantation in Montpellier (Hérault, Occitania), from a donor retrospectively classified as the first autochthonous case of WNV infection in Vaucluse department (Auvergne Rhône Alpes region), outside Occitania.

Q15 : Also Figures 5 and 7: I must presume, as not all sites included in wetland/artificial water monitoring were included in the questing mosquito surveillance, they arranged the presentation by method. However, this created some very ambiguous situations when trying to link the environmental monitoring with the questing mosquito surveillance results - at best obscuring the links. I think, if the authors truly wish to link the two conceptually (and I have a problem with that, as explained below), they try to provide a better visualization of the timings between environmental sampling and questing mosquito sampling for each site. Importantly, including sampling effort (i.e., providing test-negative sites).

R15 : Unfortunately, for logistic and financial constraints, it was impossible to install a mosquito traps at each water sampling location.

Presentation were indeed arranged by method, as indicated in the legend and the x-axis label. The indication of each department on the right side of the figure should help the reader to link the data between water monitoring and mosquito surveillance.

Also, to make the link conceptually more acceptable, we calculated the delay between the detection in mosquito surveillance and the detection in the nearest water sampling point, and created a figure (new figure 8) to picture the delay (or the anteriority) of detection in xenomonitoring and wetlands water from the closest sampling point, separated from few meter to a maximum of 1.9km.

Sampling effort and negative samples are now displayed as little black dots on the figures. A new supplementary figure was also added for 2024 campaign, with all sampling sites.

Reviewer #2:

Q16 : Page 13 L. 238. Mention the different lineage of WNV you are able to detect with your multiplex dPCR (add this information in the Material and Method section and in the result one ; explaining on how many strain of WNV you have tested your primers/probe set.

R16 : The WNV primers and probe set targeting the conserved 5'-UTR region were designed to detect Lineage 1 and Lineage 2 European lineages, including Lineages 1 and 2.

In silico analysis confirmed 100% query coverage and 100% sequence identity across 980 WNV sequences retrieved from GenBank (NC_009942.1 for lineage 1 and NC_001563.2 for lineage 2), demonstrating high conservation of this specific region across lineages. Experimentally, the performance of the multiplex dPCR was validated using reference viral RNA and synthetic targets (gBlocks) covering both Lineage 1 and Lineage 2.

We have added these details to both the Materials and Methods and Results sections (L. 236-237 in Materials and Methods and L. 359-360 in results section).

Q17 : Page 13 L. 249 and L. 253. I am not sure to understand here, why you choose 8µL for MX samples, and 4µl for water samples, based on the explanation written… Need to be more detailed (following what is explain in the discussion section?).

R17 : The choice of different template input volumes was not arbitrary but was strictly driven by the matrix-specific inhibition profiles experimentally determined and presented in Figure 3.

Environmental water samples collected from wetlands and artificial breeding sites are highly complex and typically concentrated in co-extracted PCR inhibitors (such as humic acids, salts, and organic matter). As demonstrated in our inhibition assays (Figure 3B), increasing the water template volume from 4 µL to 8 µL led to a significant drop in the final quantification of our internal process control, confirming matrix-induced PCR inhibition. Therefore, 4 µL was selected as the optimal threshold to maximize sample input while avoiding PCR impairment.

Conversely, the mosquito excreta (MX) matrix collected on aluminum foil proved to be remarkably clean and free of such inhibitory compounds. As shown in Figure 3A, increasing the MX template volume to 8 µL did not trigger any quantification decrease or signal suppression. We therefore chose to utilize the maximum possible volume (8 µL) for MX samples to optimize the analytical sensitivity of the assay, which is crucial for detecting the extremely low viral loads typically found in excreta.

We have revised the Results (L.304) sections to explicitly clarify this rationale.

Q18 : Page 15 line283. How many patented methods you have compared for nucleic acid extraction ? Only one if i understand. So modify the sentence accordingly “to the patented method.”

R18 Only a single patented extraction method was evaluated and compared against our optimized protocol. We have updated the text (L.340) to specify "the patented method" in the singular form.

Q19 : Page 16 L. 294-295. Need to have a more detailed explanation regarding this discrepancy. What is the main hypothesis regarding your result ?

R19 : We agree that the original statement was too concise. We have expanded the explanation in the Results section to clarify that the discrepancy most likely reflects the lower recovery efficiency of the initial MX extraction workflow when viral RNA was present at very low concentrations in mosquito excreta. We now specify that the optimized protocol, including improved excreta solubilization prior to nucleic acid extraction, likely increased RNA recovery and reduced extraction-related false negatives, which provides the most plausible explanation for the disappearance of this discrepancy in 2025. The hypothesis and its explanation was added to the Discussion section L. 504.

Q21 : Page 18 L. 321 and Page 19 L. 355. “Maps” with capital M.

R21 : Indeed, corrected

Q22 : Page 18 L. 334-337. Please indicate the quantity of WNV you were able to detect in your samples (as you did for USUV).

R22 : WNV viral loads were actually reported in the « 2024 operational proof of concept » section (lines 383), showing concentrations up to $2.2.10^5$ copies.mL^{-1} in mosquito excreta and $9.8.10^{2\cdot}$ copies.mL^{-1} in wetland water samples.

Furthermore, the Limits of Detection (LoD) and Quantification (LoQ) are identical for both WNV and USUV assays and detailed in the Results section under « Analytical performance » (L.318, Table 1). To avoid unnecessary redundancy, we preferred not duplicate these values in the proof of concept section.

Q23 : Page 19 L. 346-347. In figure 5, I didn’t see the double detection of WNV and USUV the 15th july neither the 17th july ? I should see an overlapping of the points and colours ? (like for 5th july Perols 1). Please modify the figure.

R23 : Indeed, one point were hiding the second one. Both are now visible and the legend was updated accordingly

Conclusions

Reviewer #1:

Q24 : The authors have to limit themselves to describing and validating a method (environmental sampling and dPCR) and demonstrating that it can be done in a seasonal field-surveillance program. They simply do not have the study design, nor the data to support any connection to spillover to mammals, nor have they provided any correlation between epizootiological parameters known to influence arbovirus transmission and their detections. There are no controls (they detected virus everywhere, apparently), the authors did not seem to consider sampling effort and test-negative data, and the data for the human/equine incidence is not clear and is problematic in terms of scale. The authors stated it precisely in the discussion

R24 : The present study had no intent to establish a causal relationship between environmental WNV/USUV detection and subsequent spillover to humans or horses, nor to quantify the epidemiological impact of this approach compared with conventional surveillance. This aspect is now explicitly stated.

This study was not limited to an analytical validation performed under laboratory conditions. It also evaluated the operational implementation of a complete environmental surveillance workflow over two consecutive transmission seasons, including site selection, repeated field sampling, collection of mosquito excreta and water, sample transport, nucleic-acid extraction, dPCR analysis, sequencing of selected positive samples, and interpretation of results within a one-week operational timeframe. To our knowledge, this constitutes an important field-based demonstration of the feasibility of detecting WNV and USUV RNA in heterogeneous environmental matrices during routine seasonal surveillance.

Regarding sampling effort and negative data, the total numbers of samples collected are now explicitly reported for 2024 (L. 380) and 2025 (L. 422 and L. 444). Negative samples are also now shown in Figures 5 and 7 for 2024 and 2025, and in Supplementary Figure 5 for the complete 2024 dataset (given the size of the corresponding figure). These data allow the reader to assess both the extent of the sampling effort and the distribution of negative and positive results. Indeed, 2025 could have had a site outside of the known area of WNV/USUV circulation as control sites. However, alongside our analycal tests showing a very low chance of false positive detections, the repeated negative results obtained over time and across sampling sites provide complementary evidence that controls were not necessary.

We also agree that the comparison with human, equine, and mosquito surveillance data could be questioned because these datasets were generated at different spatial and temporal scales and were not collected according to a common prospective study design. The topic is now addressed in the text and in R14. We also explicitly state that the observed environmental detections cannot be used to infer mammalian spillover, transmission intensity, or the absence of risk in areas without reported human or equine cases (L.592).

Q25 : L444 - they are providing a "field-compatible" validated analytical framework; however, this framework should be evaluated for its ability to improve/supplant/augment

proactive surveillance and actionable public health measures - they have not done this here. Therefore, they must remove this as an aspect of their study. for example (but not limited to):

R25 : We have retained a more cautious statement emphasizing the distinctive early-warning potential of environmental matrices. There is no direct comparison between our methodology and other surveillance system, just the comparison of a proxy of them, through the subsequent alerts. Unlike human or equine surveillance, which generally detects infection after spillover has occurred, environmental sampling may detect arboviral RNA before human or equine cases are confirmed. To our knowledge, environmental matrices currently represent the only surveillance matrices, with avian surveillance (L.541), with this potential for genuinely early detection at the interface between vectors, hosts, and the environment.

We have clarified that this is a conceptual and operational advantage, rather than an outcome formally demonstrated by the present study. Accordingly, the revised manuscript describes our approach as a field-compatible method with potential utility for early environmental surveillance, while explicitly stating that its ability to improve surveillance sensitivity, provide actionable information, or complement existing systems must be evaluated prospectively. We also reinforced the ideas about the needs of complementary approaches, as no system could supplant by itself all of the others (L.537).

Q26 : - L386 "remarkable temporal precedence decisive for efficient arbovirus surveillance" - apparently some sites (18? - in one department?) were early-season positive in wetland samples but negative for human "autochthonous cases" or "equine alerts"; some were questing mosquito trap-negative, wetland positive, but had both human cases and equine alerts.

R26 : Indeed, detection of viral material may or may not be coincidental with human or equine cases. Conversely, the surveillance methodology may have limits, failing to detect viral circulation in areas with human or equine cases. These limits and possible explanations are now addressed in the paragraph L.527-538 :

> Detection of WNV in water or mosquito excreta provides evidence of viral circulation in the environment, which may or may not coincide with human or equine cases, whether due to genuine absence of transmission to human or equine compartment, or a failure to detect infection (e.g., asymptomatic or unreported cases, misdiagnosis). Conversely, human or equine cases may occur in areas where environmental surveillance did not detect the virus. The probability of detecting WNV in water or mosquito excreta is likely influenced by several factors affecting viral input and amplification, including the abundance, community composition and infection status of avian hosts[63], as well as mosquito abundance and infection prevalence [64]. Viral persistence and detectability in environmental matrices may additionally be affected by physicochemical conditions such as temperature and solar/UV radiation [65], and finally, by the probability of sampling viral materials (e.g., the distance between infectious birds and the sampling site). These limits are inherent to any surveillance systems, underscoring the needs of complementary approaches. Nevertheless, our results demonstrate that the method is operationally sound, effective, capable of providing early detection of WNV circulation, compared to other methodologies

Q27 : - Previously, I mentioned the fact that "human cases" and "equine alerts" were not clearly defined, and seem to disagree with other publicly available data. Anyway, the authors have to consider whether the discrepancy in geographic scales prevents meaningful correlations.

R27 : Human and equine cases and alerts are now defined (L.111-115) in the Introduction: “equine or human cases” are hereinafter defined as detection of symptomatic or asymptomatic WNV/USUV cases in humans or horses, and the corresponding “alerts” refer to notifications issued through the Ministry of Health’s SI-LAV platform for human cases, or the RESPE epidemiological surveillance network (in connection with the French National Reference Laboratory, LNR) for equine cases.

We agree that differences in the geographic scales of environmental surveillance and reported human or equine cases may limit the interpretation of direct spatial correlations. However, the objective of this study was not to establish a direct spatial association between individual human or equine cases and specific sampling locations. Rather, our aim was to evaluate the operational implementation of the surveillance system and its ability to provide an early indication of WNV circulation at a scale relevant to public and veterinary health interventions.

Q28 : - L423 - the authors do not have the data to support this

R28 : The end of the sentence “[…] temporal advantage over conventional surveillance systems” was replaced by “[…] temporal advantage over conventional alert and associated surveillance systems” (L.514-515) to more accurately reflect the scope and purpose of the comparison.

Q29 : - L427 - check that publicly available data agree

R29 : The origin of non-publicly available data provided by the mandated vector control operator is now detailed in the M&M (L. 208-211)

Q30 : - L429-431 - this was not shown, rather, conflicting results were provided, both in support and in contrast

R30 : the sentence was modified to be supported by the results (also modified, see above)

Q31 : - L436 - *sometimes* occurred before detection in mosquito excreta, and the comparability of the geographic areas was variable (sometimes yes, sometimes no) and uncertain (in terms of scale).

R31 : The sentence was modified: “However, for both target viruses when viral materials were detected in both matrices, detections in environmental water but one occurred weeks before detection in mosquito excreta within the same geographic area” (L.544-546)

Q32 : - L439-440 - maybe for other viruses, but the authors did not demonstrate this for WNV/USUV here, and they should qualify this statement, because it has been shown for other viruses.

R32 : The sentence was modified to better reflect our results vs hypotheses about competitivity between viruses, and a references are now provided. L549-550

Q33 : - Actually the references in L436-442 show a troubling pattern of providing a reference for one aspect of their sentence (the fact that WNV can be detected in feathers, or two self-references of WNV genomic epidemiology), but placing it in a way to support some stretched conclusions about environmental monitoring.

R33 : Every aspects are now supported by references.

Q34 : - L457 - however, based on the data, one might have recommended widespread application of larvicides, for example, in sites/departments/regions where no (symptomatic) human case arose.

R34 : Indeed. We completed the sentence with ", avoiding more costly, untargeted extensive larvicide treatments" (L.572).

Q35 : In summary, L462 - yes, the authors did an excellent job providing a scalable, field-compatible framework for arbovirus surveillance. *What they did not do was show that this enables earlier detection or provides better use of resources as opposed to conventional surveillance programs.* Therefore the rest of this paragraph - and Figure 8 - is unnecessary, as overlooks (or exaggerates) the fact that the authors did not provide clear empirical evidence that this is "better" or "important" for surveillance programs.

R35 : We agree that these data do not, by themselves, demonstrate quantitatively that the proposed system is more effective or resource-efficient than conventional surveillance programs, now stated L.608-612. However, the delays between our environmental detections and alerts generated by conventional surveillance provide empirical evidence of the temporal precedence of our approach.

Figure 9 (formerly Fig. 8) and the accompanying paragraph aim to immediately bridge the gap between our research findings and their potential integration into field monitoring and operational decision-making, which is a common shortcoming in the operational implementation of research findings.

Q36 : It is certainly interesting! I applaud the authors for a nice manuscript - a methods paper paired with field implementation really moves the impact higher from "we designed an assay" to "we designed an assay and showed some interesting results". The outlook should be, "someone should design a study to investigate whether this is really worth the effort", and not (L470) " this framework clearly shows 'early detection of emerging Culex-borne viruses'..and 'offers unprecedented lead time to guide public health interventions'" etc etc.

R36 : We agree that a dedicated study would be valuable to quantitatively assess the added value of this approach compared with conventional surveillance systems. Such an assessment would allow the benefits and resource implications of environmental multi-compartment surveillance to be formally quantified. However, we consider that the results presented here provide sufficient empirical basis to support the broader perspective stated in L470 (now L.612), particularly regarding the demonstrated sensitivity, operational feasibility, scalability, and temporal precocity of the approach.

A sentence has been added to express the current needs for quantitative assessments (L.608), and we have toned down the phrasing.

Reviewer #2:

Q37 : Minor correction : In figure 8, Culex sp. (sp. with small letter)

R37 : Indeed, corrected

Reviewer #3:

Q38 : Conclusions are justified by the results of the study; this is one of the first REAL early warning signs that can readily be detected well before any outbreak. A potential game changer in the world of mosquito-borne disease.

R38 : We thank the reviewer for his/her enthusiasm, which we share.

Editorial and Data Presentation Modifications?

Reviewer #1: Minor editorial:

Q39 : L43, L115 - I have a problem with "breeding sites" as "breeding" does not mean the same as "habitats for development of immatures" in a scientific sense.

R39 : That is entirely true. We replaced "breeding sites" by wetlands (as adult and larval habitats, when considering potential introduction of viral material from both stages) , or larval development sites/larval habitats depending on the context.

Q40 : L56, L144 - following ICTV convention for viruses ("orthoflaviviruses" is not a taxon) and ICZN for animals (Culicidae should not be italicized)

R40 :Indeed. Both words are not italicized anymore.

Q41 : L58 - may be infected through infected mosquitoes taking blood meals

R41 : The text was revised following the reviewer's recommendation (L.60)

Q42 : L58 - "...epidemiologically incidental "dead-end" hosts..."
R42 : The word was change following the reviewer's recommendation.

Q43 : L85 - "...can originate from..."
R43 : The mistake was corrected.

Q44 : Please clarify : L26, L74... "most surveillance is based on clincial and veterinary reports" (L26,L74,). This clearly depends on region.

R44 : We have now clarified that this statement refers to the French situation.

Q45 : Please clarify : L94 "viral genomes should be detected first in water - introduced by infected bird excretion ..." Why?

R45 : We expected viral genomes to be detectable in water in early season, before the onset of mosquito activity, when infected birds (migratory and/or resident) are the only plausible source of viral genomes entering aquatic environments through excretion. Subsequently, as mosquito activity resumes and enzootic transmission cycles become established, viral genomes were expected to be detected in local mosquito populations. We have revised the corresponding sentences to clarify this rationale (L.105-108).

Q46 : Figures 4 and 6 maps, some of the dates are confusing (i.e., 30. Sept-6. Oct, 26. Aug-1.Sept).

R46 : That is totally true, the dates were confusing, if not erroneous, when the sampling week overlapped on two consecutive months. The dates are now corrected.

Q47 : Figures 5 and 7: please provide a legend for the dashed lines.

R47 : The legends are updated with more details about the dashed lines and the new features of the figures.

Reviewer #2:

Q48 : See the minor revisions, editorial suggestions, proposed in the different section of the manuscript (Introduction, Methods, Results, some Tables/Figures)

R48 : The suggestions were taken into consideration and revisions were made accordingly.

Q49 : L153 - "plagued by" is ambiguous
R49 : We changed the phrasing, replacing “plagued” by a more precise “harbouring a confirmed autochthonous human case with ” (L.189)

Summary and General Comments

Reviewer #1:

Q50 : The authors present a well-validated protocol for sampling aquatic sites and testing them for the presence of arboviruses. They show the implementation of this in a region of France where WNV and USUV are known to circulate seasonally. Their assay validation and methods are excellent. Their field work seems reasonable. Their conclusions relative to the importance of this approach (environmental sampling for arboviruses) remain preliminary, and they must remove this from their manuscript.

R50 : We thank the reviewer for recognizing the quality of the assay validation, sampling protocol, and field implementation. We agree that the conclusions regarding the epidemiological importance and public-health impact of environmental arbovirus surveillance should remain cautious and that these aspects were not formally demonstrated in the present study.

However, we respectfully consider that the environmental approach conclusion itself should not be removed from the manuscript. The primary objective was to establish and field-test a validated, operationally feasible protocol for detecting arboviral RNA in aquatic environmental matrices during seasonal circulation of WNV and USUV. The repeated detection of viral RNA under real field conditions demonstrates that this approach is technically feasible and can generate surveillance information from environmental samples.

We have therefore revised the manuscript to distinguish clearly between the demonstrated findings –analytical validation, field feasibility, and detection of WNV/USUV RNA in aquatic sites– and the potential applications, which remain preliminary and require prospective evaluation.

We have removed or softened statements suggesting that environmental sampling has already been proven to improve surveillance, predict spillover, quantify transmission, or directly guide public-health interventions. The Discussion and Conclusions now explicitly state that the epidemiological significance, added value, and operational impact of this approach remain to be determined through larger, standardized, and comparative surveillance studies.

Thus, we have retained environmental arbovirus sampling as the central contribution of the study, for the predictive and cost effective method of active surveillance of arbovirus.

Q51 : The manuscript had no essential statements/discussions of limitations. As the authors provided two seasons of implementation, they could provide an excellent scientific service by describing pitfalls/limitations in their applied methods, as they and others will surely want to test the system in scientifically controlled field experiments. At the very least, the authors should provide a more scientific discussion of the limitations of their ability to infer epidemiologic/epizootiologic outcomes based on their wastewater sampling. Surveillance programs for mosquitoes and mosquito-borne diseases have been around 100 years now.

R51 : We agree that, despite two seasons of field implementation, the manuscript did not present a consolidated discussion of the practical limitations and pitfalls of the method.

However, the epistemological limits on inferring epidemiologic and epizootiologic outcomes from environmental detections are now addressed in our responses to comments R24–R26, R35 and R36, and in the Discussion paragraph beginning “Detection of WNV in water or mosquito excreta provides evidence of viral circulation…”. We have also added a dedicated paragraph to the Discussion (L. 568-588), before the Conclusion, consolidating these points and adding the methodological pitfalls identified through field implementation, including the analytical limitations (physicochemical heterogeneity, amplicon size), the logistics and protocol avenues of improvement, and the confidentiality obstacles to fine-scale spatial inference with human and equines cases. The cost-effectiveness of the method is also discussed above.

Q52 : The authors have made a good case that this method should be assessed relative to traditional methods - and the biological considerations about the source of the environmental virus "signal" should receive some more work - but this manuscript comes no where near addressing the topic and these limitations should be addressed. At the expense of being repetitive, the field implementation provided interesting results, but the main limitation was that it did not provide clear links between conventional surveillance (human cases, "equine alerts", detection in questing mosquitoes), and, in fact, was not designed to show these links as a preliminary study.

R52 : We acknowledge the reviewer’s point and would like to clarify the objective of the present study. The purpose of our implementation experiment was not to establish direct links between individual conventional surveillance outcomes (human cases, equine alerts, or virus detection in questing mosquitoes) and related environmental detections. Our objective was to assess whether the proposed environmental surveillance system could detect viral circulation sufficiently early to provide an operational signal preceding the alerts generated by conventional surveillance.

From this perspective, the relevant comparison is the timing of environmental detection relative to the subsequent triggering of conventional alerts, at the relevant operational scale. The only unaddressed temporal component in the present study is the time required to translate an environmental detection into an operational alert. Establishing direct epidemiological or spatial links between individual conventional surveillance events and environmental viral signals would require additional data and a dedicated study and is therefore beyond the scope of the present work.

Reviewer #2:

Q53 : To conclude, this manuscript is a very well written, conclusions and the novel method developed are supported by the results.
The new method developed, multiplex dPCR, is very specific and sensitive and allow an accurate detection of WNV and USUV. The novelty of the work, is the development of nucleic acid extraction of water samples and excreta samples, allowing the detection of WNV/USUV with the dPCR developed.

This work represent an innovative approach to be able to improve surveillance of WNV/USUV in environmental samples, allowing an earlier detection of WNV/USUV circulation and a better evaluation of the risk of emergence of human/equine cases.

R53 : We sincerely thank the Reviewer for this highly positive assessment. Our results demonstrate that the multiplex dPCR assay and the optimized extraction procedures provide a sensitive, specific, and field-compatible approach for detecting WNV and USUV RNA in water and excreta samples.

This study opens a new avenue for environmental surveillance by enabling the detection of viral circulation before, or alongside, the identification of human, equine, or mosquito cases. Although further prospective validation is warranted, the consistent environmental detections observed across two seasons support the potential predictive value of this approach for anticipating periods of increased emergence risk and strengthening integrated WNV/USUV surveillance.

Figure resubmission:

While revising your submission, we strongly recommend that you use PLOS's NAAS tool (https://ngplosjournals.pagemajik.ai/artanalysis) to test your figure files. NAAS can convert your figure files to the TIFF file type and meet basic requirements (such as print size, resolution), or provide you with a report on issues that do not meet our requirements and that NAAS cannot fix.

After uploading your figures to PLOS's NAAS tool
- https://ngplosjournals.pagemajik.ai/artanalysis, NAAS will process the files provided and display the results in the "Uploaded Files" section of the page as the processing is complete. If the uploaded figures meet our requirements (or NAAS is able to fix the files to meet our requirements), the figure will be marked as "fixed" above. If NAAS is unable to fix the files, a red "failed" label will appear above. When NAAS has confirmed that the figure files meet our requirements, please download the file via the download option, and include these NAAS processed figure files when submitting your revised manuscript.

Reproducibility:

To enhance the reproducibility of your results, we recommend that authors of applicable studies deposit laboratory protocols in protocols.io, where a protocol can be assigned its own

identifier (DOI) such that it can be cited independently in the future. Additionally, PLOS ONE offers an option to publish peer-reviewed clinical study protocols. Read more information on sharing protocols at https://plos.org/protocols?utm_medium=editorial-email&utm_source=authorletters&utm_campaign=protocols
