# Supplementary material for: Early-warning surveillance of West Nile and Usutu viruses in wetland and larval habitat waters and in mosquito excreta using digital PCR
Source: PLoS Negl Trop Dis. 2026 Oct 6;20(10):e0014799. doi: 10.1371/journal.pntd.0014799 (PMC13657283; doi:10.1371/journal.pntd.0014799)
Supplement: Supplementary file 2 [file pntd.0014799.s005.docx]

| **S2 Table. Primers and probe targeting WNV and USUV viruses** | | | | |
| --- | --- | --- | --- | --- |
| Target | Oligonucleotids | Amplicon size (bp) | Sequence accession number | Reference |
| WNV | F 5' CTGTGTGAGCTGACAAACTTA 3' | 105 | Lineage 1 NC_009942.1 Lineage 2 NC_001563.2 | (Kolodziejek et al., 2014) |
|  | P 5' TGCGAGCTGTTTCTTAGCACGA 3' |  |  |  |
|  | R 5' CTCCTGGTTTCTTAGACATCG 3' |  |  |  |
| USUV | F 5' CATCGTTCTCGACTTTGACT 3' | 88 | NC_006551 | (Jöst et al., 2011) |
|  | P 5' ACCGTCACAATCACTGAAGCAT 3' |  |  |  |
|  | R 5' AGTAGTTCTTATGGAGGGTCC 3' |  |  |  |
