# Supplementary material for: Early-warning surveillance of West Nile and Usutu viruses in wetland and larval habitat waters and in mosquito excreta using digital PCR
Source: PLoS Negl Trop Dis. 2026 Oct 6;20(10):e0014799. doi: 10.1371/journal.pntd.0014799 (PMC13657283; doi:10.1371/journal.pntd.0014799)
Supplement: Supplementary file 3 [file pntd.0014799.s007.docx]

| **S4 Table. Stability test Pan-mosquito and WNV viruses over time** | | | | | | |
| --- | --- | --- | --- | --- | --- | --- |
| Sample | Matrix | Municipality | Sampling date | Shelf life at 4°C(days) | [Pan-mosquito] (Copies/L) | [WNV] (Copies.L) |
| 1 | Water - Artificial breeding site | Baillargues, France | 09/09/2024 | 0 | 1,62E+10 | 1,25E+05 |
| 1 | Water - Artificial breeding site | Baillargues, France | 09/09/2024 | 8 | 1,48E+10 | 2,47E+05 |
| 1 | Water - Artificial breeding site | Baillargues, France | 09/09/2024 | 15 | 1,33E+08 | 3,56E+05 |
| 1 | Water - Artificial breeding site | Baillargues, France | 09/09/2024 | 23 | 6,12E+06 | 1,09E+05 |
| 1 | Water - Artificial breeding site | Baillargues, France | 09/09/2024 | 25 | 2,40E+09 | 5,52E+04 |
| 1 | Water - Artificial breeding site | Baillargues, France | 09/09/2024 | 28 | 1,76E+06 | ND |
| 1 | Water - Artificial breeding site | Baillargues, France | 09/09/2024 | 42 | 1,40E+06 | ND |
| 2 | Water - Wetlands | Baillargues, France | 09/09/2024 | 0 | 1,46E+10 | 6,38E+04 |
| 2 | Water - Wetlands | Baillargues, France | 09/09/2024 | 8 | 7,28E+09 | 5,63E+04 |
| 2 | Water - Wetlands | Baillargues, France | 09/09/2024 | 15 | 4,57E+09 | ND |
| 2 | Water - Wetlands | Baillargues, France | 09/09/2024 | 15 | 4,06E+09 | 1,12E+05 |
| 2 | Water - Wetlands | Baillargues, France | 09/09/2024 | 23 | 1,31E+08 | 1,10E+05 |
| 2 | Water - Wetlands | Baillargues, France | 09/09/2024 | 25 | 5,79E+07 | 1,12E+05 |
| 2 | Water - Wetlands | Baillargues, France | 09/09/2024 | 28 | 4,01E+07 | 5,83E+04 |
| 2 | Water - Wetlands | Baillargues, France | 09/09/2024 | 42 | 2,52E+07 | ND |
