# Supplementary material for: Early-warning surveillance of West Nile and Usutu viruses in wetland and larval habitat waters and in mosquito excreta using digital PCR
Source: PLoS Negl Trop Dis. 2026 Oct 6;20(10):e0014799. doi: 10.1371/journal.pntd.0014799 (PMC13657283; doi:10.1371/journal.pntd.0014799)
Supplement: Supplementary file 4 [file pntd.0014799.s008.docx]

| S5 Table. Sequencing by Sanger | | | | |
| --- | --- | --- | --- | --- |
| Sampling date | Municipality | Accession number | Primers used | Sequences obtained by Sanger sequencing |
| 09/09/2024 | Baillargues (10) | PZ530941 | R2 | AAAAGGTTCTGTGTGAGCTGACAAACTTAGTAGTGTTTGTGAGGATTAACAACAATAACACAG |
| 09/04/2025 | La Palme (37) | PZ530943 | R1 | GATGACAACATTAACACAGTGCGAGCTGTTTCTTAGCACGAAGATCTCGATGTCTAAGAAACCAGGTGTAG |
|  |  |  | R2 | GGTTTTCTGTGTGAGCTGACAAACTTAGTAGTGTTTGTGAGGATTAACAACAATTAACACAGTGCGAGTCTGTTG |
| 09/04/2025 | Narbonne (44) | PZ530944 | R1 | GGACATTAACACAGTGCGAGCTGTTTCTTAGCACGAAGATCTCGATGTCTAAGAAACCAGGTGAACT |
|  |  |  | R2 | TTTTCTCTGTGTGAGCTGACAAACTTAGTAGTGTTTGTGAGGATTAACAACAATTAACACAGGGCGATC |
| 09/04/2025 | Fleury (34) | PZ530942 | R1 | ACACATTAACACAGTGCGAGCTGTTTCTTAGCACGAAGATCTCGATGTCTAAGAAACCAGGTAGA |
|  |  |  | R2 | GCCAATTCTGTGTGAGCTGACAAACTTAGTAGTGTTTGTGAGGATTAACAACAATAACAC |
