# Supplementary material for: Early-warning surveillance of West Nile and Usutu viruses in wetland and larval habitat waters and in mosquito excreta using digital PCR
Source: PLoS Negl Trop Dis. 2026 Oct 6;20(10):e0014799. doi: 10.1371/journal.pntd.0014799 (PMC13657283; doi:10.1371/journal.pntd.0014799)
Supplement: Supplementary file 5 [file pntd.0014799.s009.docx]

**S1 Text: Method development for nucleic acid extraction from MX samples**

An initial in-house protocol was used for nucleic acid extraction from mosquito excreta (MX) samples, consisting of manually collecting mosquito excreta with a loop, resuspending them in 0.2 mL of phosphate-buffered saline (PBS, pH 8), and directly extracting the entire volume using the Indimag Pathogen kit (Indical Bioscience, Germany). The optimized protocol adapted from Bigeard et al. (Bigeard et al., 2024) (hereafter referred to as the “literature protocol”) was developed to improve excreta recovery. The aluminium foil from the MX adapter was coiled and placed in a 50 mL plastic tubes (Greiner Bio-one, Cat. No.227261), soaked in 1.2 mL VXL lysis buffer (Cat. No.1069974, Qiagen, Germany) and vortexed for 5 min. The supernatant was recovered, and 0.2 mL were used for nucleic acid extraction using Indimag Pathogen Kit (Cat. No. SP947457, Indical bioscience, Germany). Nucleic acid were eluted in 0.1 mL H_2_O.

The performance of both protocols was compared using mosquito excreta artificially deposited on aluminium foil and spiked with USUV at three concentrations: Low (4.6 × 10² TCID₅₀.mL^-1^), Medium (4.6 × 10³ TCID₅₀.mL^-1^) and High (4.6 × 10⁴ TCID₅₀.mL^-1^). Each concentration level was prepared in duplicate. Viral detection and quantification were performed by dPCR and results were compared across protocols. The pan-mosquito target was used as an extraction control to assess nucleic acid recovery efficiency, while USUV quantification reflected viral detection performance.

**Figure A: Digital-PCR and acid nucleic extraction methods optimization from mosquito excreta.** dPCR inhibition assays were conducted from a side-by-side analysis (‘literature’ protocol described in Bigeard et al. (2024), vs the in-house, alternative one) on three USUV-spiked MX samples at 3 different concentrations (Low : 4.6**× 10**^2^ TCID_50_**.mL⁻¹**, Medium : 4.6**× 10**^3^ TCID_50_**.mL⁻¹**, and High: 4.6**× 10**^4^ TCID_50_**.mL⁻¹**). Y-axis is in logarithmic scale log10(x+1).

Based on these comparisons, the protocol adapted from the literature consistently yielded higher pan-mosquito and USUV signals across comparable spiking levels (Fig. A), indicating improved extraction and detection efficiency. This protocol was therefore selected for the analysis of all MX samples from the 2024 campaign and for subsequent field applications in 2025. To further improve excreta solubilisation, the lysis buffer volume was increased from 0.8 mL to 1.2 mL, and this modified version was retained for routine processing.
