# Supplementary material for: Early-warning surveillance of West Nile and Usutu viruses in wetland and larval habitat waters and in mosquito excreta using digital PCR
Source: PLoS Negl Trop Dis. 2026 Oct 6;20(10):e0014799. doi: 10.1371/journal.pntd.0014799 (PMC13657283; doi:10.1371/journal.pntd.0014799)
Supplement: Supplementary file 6 [file pntd.0014799.s010.docx]

**S2 Text: Method development for nucleic acid extraction from wetlands and artificial site water samples**

The water samples, with volumes comprised between 0.1 to 1 L, were kept at 4°C after sampling until nucleic acid extraction. Extractions of total DNA/RNA for dPCR analyses were performed following the IAGE patented method (Durandet et al., 2022) with an optimized pre-treatment. Briefly, the patented method consists in thoroughly mixing the total volume of water, sub-sampling and applying 15 mL from each homogenized sample to an Amicon® Ultra-15 Centrifugal Filter Unit (cut-off: 10 kDa, Cat. No. UFC901096, Merck Millipore, Germany), previously hydrated with 2.5 mL of MQ water and spin for 10 min at 3234 g, at 4°C. The amicon was then centrifuged for 35 minutes at 3234 g, at 4°C. If the resulting volume was inferior to 0.5 mL, the tube was centrifuged again for 10 minutes at 3234 g, at 4°C.

The optimized version added a centrifugation of the 15 mL sub-sampling for 15 min at 3234 g at 4°C. For each sample, pellet was conserved on ice until further treatment. Supernatant was recovered and applied to an Amicon® Ultra-15 Centrifugal Filter Unit (cut-off: 10 kDa, Cat. No. UFC901096, Merck Millipore, Germany) as for the patented method. The resulting volume of the concentration was used to resuspend the corresponding pellet for each sample. Then, 0.4 mL of Nucleo Protect VET Buffer (Cat. No. 740750, Macherey Nagel, Germany) was added before grinding with two 3-mm stainless steel beads on a Geno/Grinder ® instrument (Spex Sample Prep LLC, USA) for 15 s at 1500 cpm, thrice. In both protocols, extractions were then performed from 0.2 mL using the Indimag Pathogen kit (Cat. No. SP947457, Indical Bioscience, Germany) and nucleic acid were then eluted in 0.1 mL H_2_O.

To compare the patented method with the optimized version, two water samples and two larval habitat water samples were spiked with USUV at a concentration of 1.10⁷ copies·L⁻¹. Each sample underwent both extraction protocols. Additionally, one non-spiked sample from the Zoological park of Montpellier was extracted in triplicates using both methods; only pan-mosquito concentration, was quantified and used as a positive control for comparison. All samples, from 2024-proof of concept and 2025-field trial, were subsequently analysed with the optimized version.

Because such sampling campaigns may require reanalysis of previously analysed samples, and given the complexity of water as a matrix, we evaluated the stability of viral targets during raw water storage. Thus, we assessed whether storage at 4°C reliably preserve viral RNA and determined the maximum duration over which this stability could be ensured. Two WNV-positive samples–one from wetlands and one artificial site water– were stored at 4°C and re-extracted at 8, 15, 23, 25 and 28 days post- collection.
