# Supplementary material for: Early-warning surveillance of West Nile and Usutu viruses in wetland and larval habitat waters and in mosquito excreta using digital PCR
Source: PLoS Negl Trop Dis. 2026 Oct 6;20(10):e0014799. doi: 10.1371/journal.pntd.0014799 (PMC13657283; doi:10.1371/journal.pntd.0014799)
Supplement: Supplementary file 7 [file pntd.0014799.s011.docx]

**S3 Text: Digital-PCR method characterization and quality control set-up**

To assess the ability of primers and probes to specifically target WNV and USUV, RT-dPCR amplification was conducted on RNA extract from both viruses. Non-target viruses were also chosen for specificity assays : (i) WNV and USUV to test cross-reactivity between both targets, (ii) other Arboviruses as dengue (DENV-1, DENV-2, DENV- and DENV-4), Chikungunya and Zika viruses, (iii) viruses commonly monitored in water-based surveillance as Hepatitis A virus (HAV), Measles virus (Wild-type strain), Respiratory syncytial virus A and B (RSV-A and B), SARS-CoV-2, Influenza A virus, H5N1 avian influenza virus and Norovirus GI/GII.

A multiplex assay was developed to quantify WNV and USUV in the same analysis. Two other targets were incorporated in the multiplex as controls. First, a multispecies mosquito probe (hereafter called “pan-mosquito”; designed from a consensus sequence of 146 ITS1 sequences from genera *Aedes*, *Culex* and *Anopheles*). Then, a system targeting a random exogenous RNA that was added in small amount to each sample in order to determine the presence of inhibitors (hereafter called “inhibition control”). Positive and negative controls of dPCR were also added to analysis. Digital-PCR reactions were conducted using a multiplex assay on a QIAcuity® Eight Plateform System (Qiagen, Germany), using the QIAcuity ® One-Step Viral RT-PCR Kit (Cat. No. 210212) and QIAcuity Nanoplate 26 K 24-wells (Cat. No. 250001). The dPCR reaction mixtures were prepared in a preplate as follows. For each reaction, 10 µL of 4 × One-Step Viral RT-PCR Master Mix, 0.4 μL of 100 × Multiplex Reverse Transcription Mix, 1 μL of the primers/probes mix (final concentration 0.45 µM of both primers and 0.125 µM of probe), and a volume of sample nucleic acids extract, were combined with H_2_O up to 40 µL final reaction volume. Reaction mixtures were then transferred into a QIAcuity® Nanoplate and the plate was loaded onto the fully automated QIAcuity ® Eight instrument. The workflow included (i) priming and rolling step to generate and isolate the chamber partitions (26,000 partitions), (ii) amplification step under the following cycling protocol: 50 °C for 40 min for reverse transcription, 95 °C for 2 min for enzyme activation, 95 °C for 5 s for denaturation, and 58 °C for 60 s for annealing/extension for 40 cycles; and (iii) imaging step by reading fluorescence emission after excitation of the probe at the appropriate wavelength.

Samples from the 2024-campaign were first analysed using 2 µL of sample both for MX samples and water samples. This input volume was applied to all samples collected from the beginning, up to those sampled in mid-September. An optimisation of the input volumes was then performed on chosen samples (varying in term of origin) to test potential PCR inhibition and improve the representativity of the measure in the remaining samples from 2024 and 2025 campaign. For MX samples, USUV-spiked samples at different concentrations (Low: 4.6 TCID_50_.mL^-1^, Medium: 4.6 x 10^1^ TCID_50_.mL^-1^ and High : 4.6 x 10^2^ TCID_50_.mL^-1^) were prepared, extracted and tested in duplicates with three different input volumes (2, 4 and 8 µL). For water samples, artificial breeding sites were used, as they exhibited the highest levels of inhibition in preliminary tests. Two water samples (Zoological park of Montpellier and artificial breeding sites from vector control operation in Pérols, south Montpellier) were tested in triplicates using two sample input volumes (4 and 6 µL). Pan-mosquito and inhibition controls concentrations were compared depending on the sample input volume.

The smallest copy number of the target sequence detectable in a reliable manner per dPCR well (absolute limit of detection; LoD) was determined for WNV and USUV. A positive internal control was diluted to achieve final concentrations corresponding to, respectively, 5 and 10 RNA copies per dPCR reaction (copies deposited in plate wells). Each dilution was measured in ten replicates on the same dPCR run. Moreover, the smallest copy number of the target sequence from which the abundance of that sequence can be measured accurately and reliably (absolute limit of quantification; LoQ) was measured for WNV and USUV by analysing a series of positive control dilutions and identifying the lowest concentration at which quantification remained accurate and reproductible, based on inter-replicate variance and established method acceptance criteria. For this purpose, ten independents’ solutions were prepared from each positive control (WNV or USUV) to reach a final concentration of 33.33 RNA copies/µL of reaction mix for each target, and each dilution was analysed once, resulting in ten dPCR measurements per tested concentration level, performed on four different thermocyclers. The blank limits (LoB), *i.e.* the highest concentration measured in the absence of the target in the dPCR well, were determined. To characterize this “background noise” of the method, thereby ensuring no false positive could be detected, two extraction negative controls which followed the entire process of sample acid nucleic extraction were quantified in ten replicates on two different thermocyclers. For each target of the multiplex, the median was calculated from the obtained twenty concentrations (in copy.µL of dPCR reaction^-1^) and standard deviation was determined. The LoB was then calculated for each target using the formula:

LoB = median + 1.645 × sd

With sd the standard deviation, and the 1.645 factor corresponding to the 95th percentile of a normal distribution. This threshold indicates that 95% of measurements obtained from negative controls are expected to fall below the LoB, and that only 5% of values would exceed the LoB due to random variation, corresponding to a low probability of false‐positive detection.
