# Supplementary material for: Early-warning surveillance of West Nile and Usutu viruses in wetland and larval habitat waters and in mosquito excreta using digital PCR
Source: PLoS Negl Trop Dis. 2026 Oct 6;20(10):e0014799. doi: 10.1371/journal.pntd.0014799 (PMC13657283; doi:10.1371/journal.pntd.0014799)
Supplement: Supplementary file 8 [file pntd.0014799.s012.docx]

**S4 Text: Virus sequencing**

Whole-genome sequencing of the virus was performed for all MX or mosquito samples with a sufficiently high viral load (Ct < 32). PCR amplifications were conducted using eight pairs of overlapping primers targeting segments of the region of interest (Bigeard et al. 2024). Reverse transcription and amplification were carried out using the SuperScript IV One-Step RT-PCR kit (ref. 12594100, Thermo Fisher, USA). Each 25 μL reaction contained 12.5 μL of 2X Master Mix, 1.25 μL (10 μM) of each forward and reverse primer, 6.5 μL of RNase-free water, 0.5 μL of RT enzyme mix, and 3 μL of extracted genetic material. Thermal cycling conditions were as follows: 55°C for 10 min, 98°C for 2 min, followed by 40 cycles of 98°C for 10 s, 55°C for 10 s, and 68°C for 1 min 45 s, with a final extension at 68°C for 5 min. PCR products were visualized by electrophoresis on a 1% agarose gel stained with EZ-Vision DNA dye (ref. 1B1680, VWR Chemicals, USA) to verify amplification success and amplicon size.

Amplicons were then purified and sent to the *Unité des Virus Émergents* (UVE, Aix-Marseille University, France) for downstream processing. Equimolar quantities of each amplicon were pooled and quantified using a Qubit fluorometer (Thermo Fisher, USA). DNA fragmentation into ~250 bp fragments was performed with a Bioruptor sonicator (Diagenode, Belgium). Library preparation was carried out using the Ion Plus Fragment Library Kit (ref. 4471252, Thermo Fisher, USA) and the automated AB Library Builder system (ref. 4463794, Thermo Fisher, USA). The pooled libraries were quantified by qPCR to confirm equimolarity, then amplified by emulsion PCR and loaded onto an Ion Chip using the Ion Chef instrument (Thermo Fisher, USA). Sequencing was performed on the Ion Torrent S5 system (Thermo Fisher, USA), generating high-quality reads for downstream analysis. Following sequencing, raw reads were demultiplexed based on their barcode identifiers, quality-filtered, and aligned to the closest viral reference genome. Consensus sequences were assembled from well-covered regions, while poorly covered areas were masked with “N” to denote undetermined nucleotides.

In parallel to the whole-genome sequencing performed for the identification of circulating lineages, targeted Sanger sequencing was conducted on samples with low WNV signals in dPCR to confirm that the detected target corresponded to the virus. The sole purpose of this step was to confirm target sequence identity in near-threshold samples and rule out non-specific amplification or primer-dimer artefacts.

Total nucleic acid from selected samples were subjected to DNase treatment using TURBO™ DNase (ref. 10722687, Thermo Fisher, USA) to remove residual genomic DNA prior to downstream analyses. For each reaction, a 10 µL mix was prepared containing 1 µL of 10X TURBO™ DNase Buffer, 1 µL of TURBO™ DNase, 1 µL nuclease free-water and 8 µL of nucleic acid extract. Samples were mixed and incubated for **30 min at 37 °C.** DNase inactivation was performed by adding **3.6 µL of 50 mM EDTA** (final 10X dilution of the supplied 0.5 M EDTA) followed by incubation at **75 °C for 10 min.** RNA was then reverse transcribed. For each sample, a reaction volume of 20 µL was prepared, consisting of 4 µL LunaScript® RT SuperMix (ref. E3010L, New England Biolabs, USA), 4 µL nuclease-free water, and 12 µL of DNase-treated extract. Reverse transcription was performed under the following cycling conditions: 25 °C for 2 min, 55 °C for 10 min, and 95 °C for 1 min. cDNA concentration was quantified using sDNA High Sensitivity or Broad Range assays (Thermo Fisher, USA). Targeted amplification of virus was performed using the Q5® Hot Start High-Fidelity 2X Master Mix (ref. M0494S, New England Biolabs, USA) with the specific forward and reverse primers. PCR programs were adapted according to primer annealing temperatures previously optimized by dPCR. Amplicons were checked by agarose gel electrophoresis (2% TBE), visualized under UV illumination, and purified using the QIAquick PCR Purification Kit (QIAGEN, Germany). Purified amplicons were quantified by Qubit™ dsDNA BR assay, diluted to the required concentration, and prepared for Sanger sequencing using the Eurofins Mix2Seq service. For each sample, 5 µL of purified DNA (1 ng.µL^-1^) and 5 µL of either forward or reverse primer (5 pmol.µL^-1^) were submitted following provider specifications.
