# Supplementary material for: Early-warning surveillance of West Nile and Usutu viruses in wetland and larval habitat waters and in mosquito excreta using digital PCR
Source: PLoS Negl Trop Dis. 2026 Oct 6;20(10):e0014799. doi: 10.1371/journal.pntd.0014799 (PMC13657283; doi:10.1371/journal.pntd.0014799)
Supplement: Peer review history

# Peer review: Decision Letter 0

**DOI:** 10.1371/journal.pntd.0014799.r001

**Authors:** Ran Wang, David Safronetz

15 Jul 2026

PNTD-D-26-01218

Early-warning surveillance of West Nile and Usutu viruses in water and mosquito excreta using digital PCR

PLOS Neglected Tropical Diseases

Dear Dr. Mocq,

Thank you for submitting your manuscript to PLOS Neglected Tropical Diseases. After careful consideration, we feel that it has merit but does not fully meet PLOS Neglected Tropical Diseases's publication criteria as it currently stands. Therefore, we invite you to submit a revised version of the manuscript that addresses the points raised during the review process.

Please submit your revised manuscript within by Sep 13 2026 11:59PM. If you will need more time than this to complete your revisions, please reply to this message or contact the journal office at plosntds@plos.org. When you're ready to submit your revision, log on to https://www.editorialmanager.com/pntd/ and select the 'Submissions Needing Revision' folder to locate your manuscript file.

Please include the following items when submitting your revised manuscript:

* A letter that responds to each point raised by the editor and reviewer(s). You should upload this letter as a separate file labeled 'Response to Reviewers'. This file does not need to include responses to any formatting updates and technical items listed in the 'Journal Requirements' section below.

* A marked-up copy of your manuscript that highlights changes made to the original version. You should upload this as a separate file labeled 'Revised Manuscript with Track Changes'.

* An unmarked version of your revised paper without tracked changes. You should upload this as a separate file labeled 'Manuscript'.

If you would like to make changes to your financial disclosure, competing interests statement, or data availability statement, please make these updates within the submission form at the time of resubmission. Guidelines for resubmitting your figure files are available below the reviewer comments at the end of this letter.

As the corresponding author, your ORCID iD is verified in the submission system and will appear in the published article. PLOS supports the use of ORCID, and we encourage all coauthors to register for an ORCID iD and use it as well. Please encourage your coauthors to verify their ORCID iD within the submission system before final acceptance, as unverified ORCID iDs will not appear in the published article. Only the individual author can complete the verification step; PLOS staff cannot verify ORCID iDs on behalf of authors.

We look forward to receiving your revised manuscript.

Kind regards,

Ran Wang, M.D.

Academic Editor

PLOS Neglected Tropical Diseases

David Safronetz

Section Editor

PLOS Neglected Tropical Diseases

Shaden Kamhawi

co-Editor-in-Chief

PLOS Neglected Tropical Diseases

orcid.org/0000-0003-4304-636XX

Paul Brindley

co-Editor-in-Chief

PLOS Neglected Tropical Diseases

orcid.org/0000-0003-1765-0002

**Additional Editor Comments (if provided):**

**Journal Requirements:**

If the reviewer comments include a recommendation to cite specific previously published works, please review and evaluate these publications to determine whether they are relevant and should be cited. There is no requirement to cite these works unless the editor has indicated otherwise.

1) We do not publish any copyright or trademark symbols that usually accompany proprietary names, eg ©,  ®, or TM  (e.g. next to drug or reagent names). Therefore please remove all instances of trademark/copyright symbols throughout the text, including:

- ® on pages: 18, and 19.

2) Please amend your detailed Financial Disclosure statement. This is published with the article. It must therefore be completed in full sentences and contain the exact wording you wish to be published.

i) State what role the funders took in the study. If the funders had no role in your study, please state: "The funders had no role in study design, data collection and analysis, decision to publish, or preparation of the manuscript."

ii) If any authors received a salary from any of your funders, please state which authors and which funders..

If you did not receive any funding for this study, please simply state: u201cThe authors received no specific funding for this work.u201d

3) We have amended your Competing Interest statement to comply with journal style. We kindly ask that you double check the statement and let us know if anything is incorrect.

**Reviewers' Comments:**

Reviewer's Responses to Questions

**Key Review Criteria Required for Acceptance?**

As you describe the new analyses required for acceptance, please consider the following:

**Methods**

-Are the objectives of the study clearly articulated with a clear testable hypothesis stated?

-Is the study design appropriate to address the stated objectives?

-Is the population clearly described and appropriate for the hypothesis being tested?

-Is the sample size sufficient to ensure adequate power to address the hypothesis being tested?

-Were correct statistical analysis used to support conclusions?

-Are there concerns about ethical or regulatory requirements being met?

Reviewer #1: The objectives are clear as stated. The authors wished to design sensitive assays to detect and monitor the presence/absence of WNV and USUV in environmental samples. In addition to optimize sample processing - as would be implemented in a standard surveillance program - they wished to develop a multiplex RT-dPCR.

The authors provide excellent detail in the design and validation of their environmental sampling technique as well as the dPCR assay.

Their description of the field survey was adequate, although there are some minor methological items which should be addressed. As this was not a major focus of the manuscript, I have only minor suggestions for improvement.

The authors provide some important information about habitats that is unfortunately, in general, missing from many published field experiment methodologies for vector surveillance. However, they have done this in a very vague way. How did they define any of these "epidemiologically relevant" parameters? How to identify an "avian biodiversity hotspot" (L118, L161) or "bird-dense" waters (Fig8)? For mosquito trapping, I think it is important to provide a minimum description of sampled habitats, but the authors should take care when using such vague terminology for specific parameters. They do not seem to be "predefined" (L139, were these the same criteria used to define environmental sampling?) They don't necessarily need to provide this to any great level of detail (site-specific parameters), as description of multiple sampling sites across a regional scale would be burdensome and would not add a lot to the manuscript. However, it is an important limitation of their ability to infer the predictive power of their surveillance schema. Therefore the authors should consider using less vague terminology, and should absolutely address the fact that their implementation experiment was not designed to show how useful this system can/could be, rather that it can be implemented on a wide geographic scale.

L143 - the authors must note that BG-Mozzibait is not intended for use with ornithophilic Culex species.

Reviewer #2: The authors have developed a new efficient method (Multiplex dPCR) able to detect WNV and UVUV in different environmental matrices in order to anticipate/alert the risk of emergence of human or equine cases.

The methods are clearly exposed and detailed. The study design is appropriate to address the objectives.

There is no further experiment/analyses to add to confirm the findings.

Nevertheless some minor corrections are needed in this section:

Introduction:

page 4 line 63: Correct "to date, no human antiviral treatments or vaccines exist for both viruses."

page 5 line 75-79: The paragraph presented 3 major ideas but they need to be more connected, linked to each other. Please rephrase this paragraph to linked it with surveillance improvment.

page 5 line 88-91 : Add a reference explaining in what situation (of which virus) it has been a good way/method of surveillance ? (For which virus this technic has allowed an efficient surveillance /anticipation of the risk of emergence)

Methods:

Page 7 line 110-111 : I was not able to find those results dealing with the confirmation of presence of the virus in the Culex females using qRT-PCR ? In which tables those results are presented ? Those results deserve to be presented, as they will allow the confirmation of the sequencing results.

Page 7 line 120: Correct “history of WNV/USUV circulation, sites with confirmed human or animal cases…”

Page 9 line 157: Suppress “ca” after covering , replace by “approximately “ ?

Page 10 line 179: Add the volume of VXL lysis buffer you use (even if this is presented in the supplemental data). Also add the information regarding at which temperature did you process your samples

Page 11 line 197: It is important to mention what “exogenous RNA inhibition control” you have use for your test. I was not able to find this information in the supplemental data neither

Reviewer #3: This study is designed very well, and carried out even better, as far as I can tell. Its conclusions are based on sound measurements with a very novel technique that, if proving reproduceable in other studies, will fundamentally change the early warning of mosquito-transmitted avian viruses.

**********

**Results**

-Does the analysis presented match the analysis plan?

-Are the results clearly and completely presented?

-Are the figures (Tables, Images) of sufficient quality for clarity?

Reviewer #1: L229-288: it is clear that this is a methods manuscript, which they confirmed in the discussion (e.g., L387). The authors provided excellent detail in the validation of the sample processing and RT-dPCR methods.

L296, L400-401. Sanger sequencing an amplicon from a Taqman RT-qPCR assay is not a good laboratory practice, particularly in diagnostics. Their results (Supplemental table) are unconvincing, and, frankly, I cannot believe they submitted these to GenBank (as the database suffers from poor quality unverified sequences). I would remove these from the manuscript (and rescind the submission to the database, if possible). Aligning the sequences provided in the manuscript to publicly available sequences does not provide an "unambiguous" (L299) identification.

Figures 5 and 7: these dashed lines are suspicious, as they seem to represent very different geographic scales than the results of trapping - making this figure ambiguous, at best, and misleading, at worst. The authors have provided links for the data, but the links do not count as "publicly available" if they are require a login. Publicly available data from Santé publique France list Week 29 (July 14.-20.) as the first autochthonous human case of WNV in Occitanie (in Hérault) in 2025 - this differs from what is shown in the figure and in the text (L372).

Also Figures 5 and 7: I must presume, as not all sites included in wetland/artificial water monitoring were included in the questing mosquito surveillance, they arranged the presentation by method. However, this created some very ambiguous situations when trying to link the environmental monitoring with the questing mosquito surveillance results - at best obscuring the links. I think, if the authors truly wish to link the two conceptually (and I have a problem with that, as explained below), they try to provide a better visualization of the timings between environmental sampling and questing mosquito sampling for each site. Importantly, including sampling effort (i.e., providing test-negative sites).

Reviewer #2: Results are clearly presented and completely presented

Figures and Tables presented sufficient quality

Supplemental figures and tables are important and necessary to confirm all the results

Nevertheless some minor revisions are needed in this section:

Page 13 line 238. Mention the different lineage of WNV you are able to detect with your multiplex dPCR (add this information in the Material and Method section and in the result one ; explaining on how many strain of WNV you have tested your primers/probe set.

Page 13 line 249 and line 253. I am not sure to understand here, why you choose 8µL for MX samples, and 4µl for water samples, based on the explanation written… Need to be more detailed (following what is explain in the discussion section?).

Page 15 line283. How many patented methods you have compared for nucleic acid extraction ? Only one if i understand. So modify the sentence accordingly “to the patented method.”

Page 16 line 294-295. Need to have a more detailed explanation regarding this discrepancy. What is the main hypothesis regarding your result ?

Table 2 : The size of the amplicon of sanger sequencing is really really small (less than 80nt), and from my point of view this is problematic for a confirmation step… Moreover I was not able to find the primers you use for this confirmation ? It seems it was the one of the dPCR ? From my point of view, when you want to confirm a new method of detection, you should confirm your results with other “recognized methods/published ones” like the WGS used for highly positive samples… For low positive samples, classical nested PCR should have been used, a pan flavivirus one, to be able to detect and confirm your findings… If this was done without success, it should be mentioned in the manuscript.

Page 18 line 321. “Maps” with capital M.

Page 18 line 334-337. Please indicate the quantity of WNV you were able to detect in your samples (as you did for USUV).

Page 19 line 346-347. In figure 5, I didn’t see the double detection of WNV and USUV the 15th july neither the 17th july ? I should see an overlapping of the points and colours ? (like for 5th july Perols 1). Please modify the figure.

Page 19 line 355. “Maps” with capital M.

Reviewer #3: Very well done, clear, nothing to add

**********

**Conclusions**

-Are the conclusions supported by the data presented?

-Are the limitations of analysis clearly described?

-Do the authors discuss how these data can be helpful to advance our understanding of the topic under study?

-Is public health relevance addressed?

Reviewer #1: The authors have to limit themselves to describing and validating a method (environmental sampling and dPCR) and demonstrating that it can be done in a seasonal field-surveillance program. They simply do not have the study design, nor the data to support any connection to spillover to mammals, nor have they provided any correlation between epizootiological parameters known to influence arbovirus transmission and their detections. There are no controls (they detected virus everywhere, apparently), the authors did not seem to consider sampling effort and test-negative data, and the data for the human/equine incidence is not clear and is problematic in terms of scale. The authors stated it precisely in the discussion L444 - they are providing a "field-compatible" validated analytical framework; however, this framework should be evaluated for its ability to improve/supplant/augment proactive surveillance and actionable public health measures - they have not done this here.

Therefore, they must remove this as an aspect of their study. for example (but not limited to):

- L386 "remarkable temporal precedence decisive for efficient arbovirus surveillance" - apparently some sites (18? - in one department?) were early-season positive in wetland samples but negative for human "autochthonous cases" or "equine alerts"; some were questing mosquito trap-negative, wetland positive, but had both human cases and equine alerts.

- Previously, I mentioned the fact that "human cases" and "equine alerts" were not clearly defined, and seem to disagree with other publicly available data. Anyway, the authors have to consider whether the discrepancy in geographic scales prevents meaningful correlations.

- L423 - the authors do not have the data to support this

- L427 - check that publicly available data agree

- L429-431 - this was not shown, rather, conflicting results were provided, both in support and in contrast

- L436 - *sometimes* occurred before detection in mosquito excreta, and the comparability of the geographic areas was variable (sometimes yes, sometimes no) and uncertain (in terms of scale).

- L439-440 - maybe for other viruses, but the authors did not demonstrate this for WNV/USUV here, and they should qualify this statement, because it has been shown for other viruses.

- Actually the references in L436-442 show a troubling pattern of providing a reference for one aspect of their sentence (the fact that WNV can be detected in feathers, or two self-references of WNV genomic epidemiology), but placing it in a way to support some stretched conclusions about environmental monitoring.

- L457 - however, based on the data, one might have recommended widespread application of larvicides, for example, in sites/departments/regions where no (symptomatic) human case arose.

In summary, L462 - yes, the authors did an excellent job providing a scalable, field-compatible framework for arbovirus surveillance. *What they did not do was show that this enables earlier detection or provides better use of resources as opposed to conventional surveillance programs.* Therefore the rest of this paragraph - and Figure 8 - is unnecessary, as overlooks (or exaggerates) the fact that the authors did not provide clear empirical evidence that this is "better" or "important" for surveillance programs.

It is certainly interesting! I applaud the authors for a nice manuscript - a methods paper paired with field implementation really moves the impact higher from "we designed an assay" to "we designed an assay and showed some interesting results". The outlook should be, "someone should design a study to investigate whether this is really worth the effort", and not (L470) " this framework clearly shows 'early detection of emerging Culex-borne viruses'..and 'offers unprecedented lead time to guide public health interventions'" etc etc.

Reviewer #2: The conclusions are supported by the data and well discussed according to available literature

Public Health relevance is well explainµ.

Minor correction : In figure 8, Culex sp. (sp. with small letter)

Reviewer #3: Conclusions are justified by the results of the study; this is one of the first REAL early warning signs that can readily be detected well before any outbreak. A potential game changer in the world of mosquito-borne disease.

**********

**Editorial and Data Presentation Modifications?**

Use this section for editorial suggestions as well as relatively minor modifications of existing data that would enhance clarity. If the only modifications needed are minor and/or editorial, you may wish to recommend “Minor Revision” or “Accept”.

Reviewer #1: Minor editorial:

L43, L115 - I have a problem with "breeding sites" as "breeding" does not mean the same as "habitats for development of immatures" in a scientific sense.

L56, L144 - following ICTV convention for viruses ("orthoflaviviruses" is not a taxon) and ICZN for animals (Culicidae should not be italicized)

L58 - may be infected through infected mosquitoes taking blood meals

L58 - "...epidemiologically incidental "dead-end" hosts..."

L85 - "...can originate from..."

L153 - "plagued by" is amibuous

Request clarification:

Please clarify : L26, L74... "most surveillance is based on clincial and veterinary reports" (L26,L74,). This clearly depends on region.

Please clarify : L94 "viral genomes should be detected first in water - introduced by infected bird excretion ..." Why?

Figures 4 and 6 maps, some of the dates are confusing (i.e., 30. Sept-6. Oct, 26. Aug-1.Sept).

Figures 5 and 7: please provide a legend for the dashed lines.

Reviewer #2: See the minor revisions, editorial suggestions, proposed in the different section of the manuscript (Introduction, Methods, Results, some Tables/Figures)

Reviewer #3: (No Response)

**********

**Summary and General Comments**

Use this section to provide overall comments, discuss strengths/weaknesses of the study, novelty, significance, general execution and scholarship. You may also include additional comments for the author, including concerns about dual publication, research ethics, or publication ethics. If requesting major revision, please articulate the new experiments that are needed.

Reviewer #1: The authors present a well-validated protocol for sampling aquatic sites and testing them for the presence of arboviruses. They show the implementation of this in a region of France where WNV and USUV are known to circulate seasonally. Their assay validation and methods are excellent. Their field work seems reasonable. Their conclusions relative to the importance of this approach (environmental sampling for arboviruses) remain preliminary, and they must remove this from their manuscript.

The manuscript had no essential statements/discussions of limitations. As the authors provided two seasons of implementation, they could provide an excellent scientific service by describing pitfalls/limitations in their applied methods, as they and others will surely want to test the system in scientifically controlled field experiments. At the very least, the authors should provide a more scientific discussion of the limitations of their ability to infer epidemiologic/epizootiologic outcomes based on their wastewater sampling. Surveillance programs for mosquitoes and mosquito-borne diseases have been around 100 years now. The authors have made a good case that this method should be assessed relative to traditional methods - and the biological considerations about the source of the environmental virus "signal" should receive some more work - but this manuscript comes no where near addressing the topic and these limitations should be addressed. At the expense of being repetitive, the field implementation provided interesting results, but the main limitation was that it did not provide clear links between conventional surveillance (human cases, "equine alerts", detection in questing mosquitoes), and, in fact, was not designed to show these links as a preliminary study.

Reviewer #2: To conclude, this manuscript is a very well written, conclusions and the novel method developed are supported by the results.

The new method developed, multiplex dPCR, is very specific and sensitive and allow an accurate detection of WNV and USUV. The novelty of the work, is the development of nucleic acid extraction of water samples and excreta samples, allowing the detection of WNV/USUV with the dPCR developed.

This work represent an innovative approach to be able to improve surveillance of WNV/USUV in environmental samples, allowing an earlier detection of WNV/USUV circulation and a better evaluation of the risk of emergence of human/equine cases.

Reviewer #3: (No Response)

[NOTE: If reviewer comments were submitted as an attachment file, they will be attached to this email and accessible via the submission site. Please log into your account, locate the manuscript record, and check for the action link "View Attachments". If this link does not appear, there are no attachment files.]

**Figure resubmission:**

While revising your submission, we strongly recommend that you use PLOS’s NAAS tool (https://ngplosjournals.pagemajik.ai/artanalysis) to test your figure files. NAAS can convert your figure files to the TIFF file type and meet basic requirements (such as print size, resolution), or provide you with a report on issues that do not meet our requirements and that NAAS cannot fix.

After uploading your figures to PLOS’s NAAS tool - https://ngplosjournals.pagemajik.ai/artanalysis, NAAS will process the files provided and display the results in the "Uploaded Files" section of the page as the processing is complete. If the uploaded figures meet our requirements (or NAAS is able to fix the files to meet our requirements), the figure will be marked as "fixed" above. If NAAS is unable to fix the files, a red "failed" label will appear above. When NAAS has confirmed that the figure files meet our requirements, please download the file via the download option, and include these NAAS processed figure files when submitting your revised manuscript.

**Reproducibility:**

To enhance the reproducibility of your results, we recommend that authors of applicable studies deposit laboratory protocols in protocols.io, where a protocol can be assigned its own identifier (DOI) such that it can be cited independently in the future. Additionally, PLOS ONE offers an option to publish peer-reviewed clinical study protocols. Read more information on sharing protocols at https://plos.org/protocols?utm_medium=editorial-email&utm_source=authorletters&utm_campaign=protocols

# Author comment: Author response to Decision Letter 1

**DOI:** 10.1371/journal.pntd.0014799.r002

10 Sep 2026

Attachment Submitted filename: Responses_reviewers.pdf

# Peer review: Decision Letter 1

**DOI:** 10.1371/journal.pntd.0014799.r003

**Authors:** Ran Wang, David Safronetz

23 Sep 2026

Dear Dr Mocq,

We are pleased to inform you that your manuscript 'Early-warning surveillance of West Nile and Usutu viruses in wetland and larval habitat waters and mosquito excreta using digital PCR' has been provisionally accepted for publication in PLOS Neglected Tropical Diseases.

Before your manuscript can be formally accepted you will need to complete some formatting changes, which you will receive in a follow up email. A member of our team will be in touch with a set of requests.

Please note that your manuscript will not be scheduled for publication until you have made the required changes, so a swift response is appreciated.

IMPORTANT: The editorial review process is now complete. PLOS will only permit corrections to spelling, formatting or significant scientific errors from this point onwards. Requests for major changes, or any which affect the scientific understanding of your work, will cause delays to the publication date of your manuscript.

Should you, your institution's press office or the journal office choose to press release your paper, you will automatically be opted out of early publication. We ask that you notify us now if you or your institution is planning to press release the article. All press must be co-ordinated with PLOS.

Thank you again for supporting Open Access publishing; we are looking forward to publishing your work in PLOS Neglected Tropical Diseases.

Best regards,

Ran Wang, M.D.

Academic Editor

PLOS Neglected Tropical Diseases

David Safronetz

Section Editor

PLOS Neglected Tropical Diseases

Shaden Kamhawi

co-Editor-in-Chief

PLOS Neglected Tropical Diseases

orcid.org/0000-0003-4304-636XX

Paul Brindley

co-Editor-in-Chief

PLOS Neglected Tropical Diseases

orcid.org/0000-0003-1765-0002

***********************************************************

Reviewer's Responses to Questions

**Key Review Criteria Required for Acceptance?**

As you describe the new analyses required for acceptance, please consider the following:

**Methods**

-Are the objectives of the study clearly articulated with a clear testable hypothesis stated?

-Is the study design appropriate to address the stated objectives?

-Is the population clearly described and appropriate for the hypothesis being tested?

-Is the sample size sufficient to ensure adequate power to address the hypothesis being tested?

-Were correct statistical analysis used to support conclusions?

-Are there concerns about ethical or regulatory requirements being met?

Reviewer #1: The authors have addressed all of my concerns with the methods

Reviewer #2: The authors have answer to all my questions, and have corrected their manuscript accordingly

**********

**Results**

-Does the analysis presented match the analysis plan?

-Are the results clearly and completely presented?

-Are the figures (Tables, Images) of sufficient quality for clarity?

Reviewer #1: The authors have addressed all of my concerns with the results

Reviewer #2: The authors have answer to all my questions, and have corrected their manuscript accordingly

**********

**Conclusions**

-Are the conclusions supported by the data presented?

-Are the limitations of analysis clearly described?

-Do the authors discuss how these data can be helpful to advance our understanding of the topic under study?

-Is public health relevance addressed?

Reviewer #1: The authors have addressed all of my concerns with the conclusions. Importantly, they have more concisely and unequivocably presented their main findings, and nicely addressed some key limitations of their study.

Reviewer #2: The authors have answer to all my questions, and have corrected their manuscript accordingly

**********

**Editorial and Data Presentation Modifications?**

Use this section for editorial suggestions as well as relatively minor modifications of existing data that would enhance clarity. If the only modifications needed are minor and/or editorial, you may wish to recommend “Minor Revision” or “Accept”.

Reviewer #1: I appreciate the additional figure showing temporal correspondence between sampling methods and the improvements to the timeline figures, presentating all sampling events throughout the study.

Reviewer #2: The authors have answer to all my questions, and have corrected their manuscript accordingly

**********

**Summary and General Comments**

Use this section to provide overall comments, discuss strengths/weaknesses of the study, novelty, significance, general execution and scholarship. You may also include additional comments for the author, including concerns about dual publication, research ethics, or publication ethics. If requesting major revision, please articulate the new experiments that are needed.

Reviewer #1: (No Response)

Reviewer #2: The authors have answer to all my questions, and have corrected their manuscript accordingly

The manuscript could be accepted in his present form

**********

PLOS authors have the option to publish the peer review history of their article (what does this mean?). If published, this will include your full peer review and any attached files.

If you choose “no”, your identity will remain anonymous but your review may still be made public.

**Do you want your identity to be public for this peer review?**  For information about this choice, including consent withdrawal, please see our Privacy Policy.

Reviewer #1: No

Reviewer #2: No

# Editor report: Acceptance letter

**DOI:** 10.1371/journal.pntd.0014799.r004

**Authors:** Ran Wang, David Safronetz

Dear Dr Mocq,

We are delighted to inform you that your manuscript, " Early-warning surveillance of West Nile and Usutu viruses in wetland and larval habitat waters and in mosquito excreta using digital PCR," has been formally accepted for publication in PLOS Neglected Tropical Diseases.

We have now passed your article onto the PLOS Production Department who will complete the rest of the publication process. All authors will receive a confirmation email upon publication.

The corresponding author will soon be receiving a typeset proof for review, to ensure errors have not been introduced during production. Please review the PDF proof of your manuscript carefully, as this is the last chance to correct any scientific or type-setting errors. Please note that major changes, or those which affect the scientific understanding of the work, will likely cause delays to the publication date of your manuscript. Note: Proofs for Front Matter articles (Editorial, Viewpoint, Symposium, Review, etc...) are generated on a different schedule and may not be made available as quickly.

Soon after your final files are uploaded, the early version of your manuscript will be published online unless you opted out of this process. The date of the early version will be your article's publication date. The final article will be published to the same URL, and all versions of the paper will be accessible to readers.

For Research Articles, you will receive an invoice from PLOS for your publication fee after your manuscript has reached the completed accept phase. If you receive an email requesting payment before acceptance or for any other service, this may be a phishing scheme. Learn how to identify phishing emails and protect your accounts at https://explore.plos.org/phishing.

Thank you again for supporting open-access publishing; we are looking forward to publishing your work in PLOS Neglected Tropical Diseases.

Best regards,

Shaden Kamhawi

co-Editor-in-Chief

PLOS Neglected Tropical Diseases

Paul Brindley

co-Editor-in-Chief

PLOS Neglected Tropical Diseases
